# Paramyxovirus matrix protein redirects METTL3 for dual regulation of viral replication and immune evasion

**DOI:** 10.1371/journal.ppat.1013755

**Published:** 2025-12-01

**Authors:** Takashi Okura, Yusuke Nakai, Taichi Kameya, Fuminori Mizukoshi, Hiyori Okura, Masatoshi Kakizaki, Fumihiro Kato, Yusuke Matsumoto, Yuichiro Nakatsu, Kaoru Takeuchi, Hirokazu Kimura, Makoto Takeda, Noriyuki Otsuki, Kazuya Shirato, Hideki Hasegawa, Akihide Ryo

**Affiliations:** 1 Department of Virology 3, National Institute of Infectious Diseases, Tokyo, Japan; 2 Life Science Laboratory, Technology and Development Division, Kanto Chemical Co., Inc., Kawasaki, Kanagawa, Japan; 3 Transboundary Animal Diseases Research Center, Joint Faculty of Veterinary Medicine, Kagoshima University, Kagoshima, Japan; 4 Tokiwa-Bio Inc., Tsukuba, Ibaraki, Japan; 5 Department of Health Science, Graduate School of Health Sciences, Gunma Paz University, Takasaki, Gunma, Japan; 6 Department of Microbiology, Graduate School of Medicine and Faculty of Medicine, The University of Tokyo, Tokyo, Japan; 7 Research Center for Influenza and Respiratory Viruses, National Institute of Infectious Diseases, Tokyo, Japan; Colorado State University College of Veterinary Medicine and Biomedical Sciences, UNITED STATES OF AMERICA

## Abstract

N6-methyladenosine (m6A) epitranscriptomic modifications play crucial roles in regulating both host and viral gene expression. Here, we revealed a novel mechanism by which paramyxoviruses exploit host m6A machinery to simultaneously enhance viral replication and suppress host immunity. Our results demonstrated that the viral matrix protein (M) of bovine parainfluenza virus type 3 (BPIV3) binds to the methyltransferase domain of METTL3 in the nucleus and facilitates its translocation to the cytoplasm through an exportin-1-dependent pathway. This mechanism is conserved across multiple paramyxoviruses, including human parainfluenza virus type 3, Sendai virus, Nipah virus, and measles virus, suggesting an evolutionarily conserved viral strategy. The relocated METTL3 catalyzes m6A modification at specific sites within viral nucleocapsid protein (N) mRNA, significantly enhancing its stability and protein expression. Using reverse genetics, we generated recombinant viruses harbouring mutations at these m6A acceptor sites, which exhibited markedly attenuated viral replication, confirming the critical role of these epitranscriptomic marks in the viral life cycle. Rescue experiments demonstrated that the expression of exogenous N protein partially restored the viral titer and concomitant genome/antigenome synthesis in m6A site mutant, indicating that reduced N protein abundance represents a key mechanism underlying impaired viral replication. Furthermore, M protein-mediated depletion of nuclear METTL3 significantly reduces m6A modification of host IFN-β mRNA, resulting in diminished interferon expression and compromised antiviral responses. Supporting this mechanism, infection with viruses bearing nuclear export signal mutations that prevent METTL3 cytoplasmic translocation, maintained IFN-β mRNA m6A modification and resulted in significantly elevated IFN-β expression. These findings provide direct mechanistic evidence that paramyxoviruses utilize M-driven METTL3 relocalization as a sophisticated immune evasion strategy. Our study illuminates how paramyxoviruses strategically manipulate epitranscriptomic regulation to create an environment conducive to viral propagation, thereby advancing our understanding of virus-host interactions and identifying potential targets for antiviral therapeutics.

## Author summary

In this study, we have identified a sophisticated strategy employed by paramyxoviruses to manipulate host epitranscriptomic RNA modification machinery during infection. Our research shows that viral matrix protein (M) interacts with the cellular methyltransferase METTL3 in the nucleus and facilitates its relocalization to the cytoplasm through an exportin-1-dependent pathway. This mechanism is conserved across multiple paramyxoviruses, including the human parainfluenza virus type 3, Sendai virus, Nipah virus, and measles virus, underscoring its evolutionary significance. The strategic relocalization of METTL3 serves dual purposes: it enhances viral gene expression by catalyzing N6-methyladenosine (m6A) modifications on viral transcripts, while simultaneously depleting nuclear METTL3 to reduce m6A marks on host interferon mRNAs, thereby suppressing immune responses. Using reverse genetics, we confirmed the functional importance of these modifications as mutations at m6A site significantly impaired viral replication capacity. Our findings reveal how paramyxoviruses reprogram cellular epitranscriptomic processes to create an environment conducive to viral replication. This epitranscriptomic modulation represents a previously unrecognized mechanism by which paramyxoviruses simultaneously enhance viral gene expression and evade host immunity. These insights advance our understanding of virus-host interactions and identify potential targets for developing novel antiviral therapeutics targeting this crucial interface between viral proteins and host RNA modification machinery.

## Introduction

The dynamic interplay between viruses and their hosts involves profound molecular mechanisms, many of which remain poorly understood. Viruses, as obligate intracellular parasites, critically depend on host cellular machinery for replication and dissemination [1]. Viral propagation encompasses genome replication, translation of viral proteins, assembly of viral particles, and processes that require extensive interactions with host factors at every stage of the viral life cycle [2]. Among the various host mechanisms exploited by viruses, epitranscriptomic modifications have emerged as significant regulators of viral replication and pathogenesis [3]. These chemical alterations to RNA molecules can substantially impact viral proliferation within host cells [1].

Among the various epitranscriptomic modifications, N6-methyladenosine (m6A) represents the most prevalent internal modification of messenger RNA (mRNA) in eukaryotes [4,5]. This modification, characterized by the addition of a methyl group to the N6 position of adenosine, was first discovered in the 1970s [6], but has recently garnered significant attention for its multifaceted roles in both cellular processes and viral infection [7]. The importance of m6A in the regulation of gene expression has only been fully appreciated in recent years, particularly with the development of high-throughput sequencing techniques capable of mapping m6A sites across the transcriptome [8,9]. The m6A modification significantly affects mRNA stability, translation efficiency, splicing, and nuclear export [10,11]. This dynamic epitranscriptomic mark is orchestrated by a sophisticated regulatory system involving three types of specialized proteins: “writers” that deposit the mark (e.g., METTL3, METTL14, and WTAP), “erasers” that remove it (e.g., FTO and ALKBH5), and “readers” that recognize and bind to m6A-modified RNA to mediate downstream effects (e.g., YTHDF1–3, YTHDC1–2) [10–12]. Among these regulatory proteins, METTL3, a key component of the m6A methyltransferase complex, is primarily characterized by its nuclear functions in cellular mRNA modification [13] and has emerged as a critical factor in virus-host interactions.

Paramyxoviruses, a family of enveloped negative-sense single-stranded RNA viruses, exhibit a distinctive life cycle that predominantly occurs within the cytoplasm of the host cells [14]. This cytoplasmic replication strategy distinguishes them from many other viral families and presents unique challenges and opportunities for viral propagation. Of particular interest is the formation and function of the replication complex or cytoplasmic inclusion body, which is a critical component of the paramyxovirus life cycle [15]. Upon entering the host cell, paramyxoviruses rapidly establish their replication machinery in the cytoplasm, circumventing the need for nuclear involvement [16,17]. This cytoplasmic replication is orchestrated by viral RNA-dependent RNA polymerase (RdRp), which is packaged within the virion. RdRp, encoded by the large protein (L), together with nucleocapsid protein (N) and phosphoprotein (P), forms the core of the replication complex [18]. The paramyxovirus replication complexes are primarily organized within liquid-liquid phase-separated structures known as inclusion bodies within infected cells [15]. These cytoplasmic compartments serve as concentrated microenvironments where viral RNA replication machinery converges with multiple host factors [19–21]. Despite their importance in the viral life cycle, the specific molecular interactions within these structures and the precise functions of recruited host factors remain incompletely characterized.

The cytoplasmic nature of paramyxovirus replication significantly influences host-pathogen interactions. Recent studies have indicated that paramyxoviruses have evolved mechanisms to utilize host cell methylation machinery for their RNA processing [4,22–24]. Since paramyxoviruses replicate in the cytoplasm, the cellular distribution of METTL3 appears to be altered during infection. Indeed, it has been observed that certain viral proteins can induce the relocalization of METTL3 from the nucleus to the cytoplasm [25,26]. This relocalization brings METTL3 into proximity to viral replication complexes, enabling its interaction with newly synthesized viral RNAs. However, the precise mechanism underlying METTL3 recruitment to viral RNAs remains largely elusive.

In our current study, we demonstrated that the paramyxovirus matrix protein (M) binds to METTL3 in the nucleus and recruits it to the cytoplasmic viral replication complex. We further revealed that redirected METTL3 binds to viral mRNA and catalyzes several m6A modifications that enhance viral replication. Furthermore, we demonstrated that this forced cytoplasmic relocalization of METTL3 depletes nuclear METTL3, significantly reducing the m6A modification of interferon-β mRNA, thereby suppressing host innate immune responses. This dual mechanism represents a sophisticated viral strategy that has been conserved across various paramyxovirus family members. The results of this study provide critical insights into the mechanistic details of how paramyxoviruses exploit the host m6A epitranscriptomic machinery, and may inform the development of novel therapeutic interventions targeting this crucial virus-host interaction.

## Results

### m6A modification is associated with BPIV3 replication in infected cells

**Fig 1:**
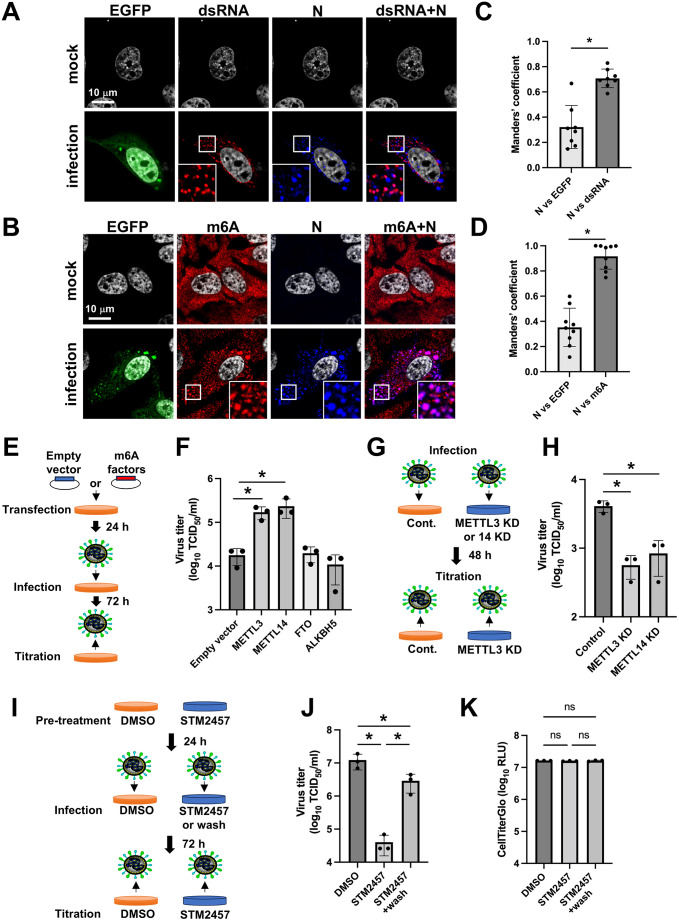
m6A modification of viral RNA during BPIV3 infection and enhancement of viral replication by METTL3. HeLa cells were infected with rBPIV3-EGFP at an multiplicity of infection (MOI) of 1 and fixed at 48 h post-infection (hpi). The cells were costained with anti-BPIV3-N antibody (Ab) and anti-double-strand RNA (dsRNA) Ab (A) or m6A Ab (B). Representative images from three independent experiments are shown. White boxes in each panel indicate regions shown as magnified views in the corresponding lower-right insets. Cell nuclei were stained with DAPI. Manders’ colocalization coefficient for N with EGFP and with dsRNA (C), or with EGFP and with m6A (D), was calculated using the Coloc 2 plugin in Fiji/ImageJ software, based on analyses of eight cells for panel C and nine cells for panel D. The 293T cells were transfected with empty vector, METTL3, METTL14, ALKBH5, or FTO expression plasmids as m6A factors, and at 24 h post-transfection, the cells were infected with rBPIV3-EGFP at an MOI of 1. At 72 hpi, the culture supernatant was harvested, and the viral titer was determined by the TCID_50_ method (E, F). METTL3-KD, METTL14-KD, or control-sh HeLa cells were infected with rBPIV3-EGFP at an MOI of 1. At 48 hpi, supernatants from infected cells were harvested, and viral titers in each cell line were determined using the TCID_50_ method (G, H). HeLa cells were pre-treated with 7.5 µM STM2457 24 h before infection, and then the cells were infected with rBPIV3-EGFP at an MOI of 1 in the presence or absence (STM2457 + wash) of STM2457. At 72 hpi, supernatants from infected cells were harvested, and viral titers were determined in each cell line using the TCID_50_ method (I, J). CelliterGlo was used to measure the cell viability of the STM2457-treated-infected cells (K). RLU: relative luminescence unit. Data are representative of three independent experiments (n = 3). Asterisks indicate significance (*p < 0.05); ns, not significant. Data represent the mean ± SD from n = 3 independent experiments. Schematic illustrations in panels E, G, and I were created with MS PowerPoint.

When paramyxoviruses infect cells, viral RNA (vRNA) and viral mRNAs are synthesized in inclusion bodies that serve as replication complexes [15]. To investigate whether vRNA undergoes m6A modification within these structures, we infected HeLa cells with recombinant bovine parainfluenza virus type 3 expressing enhanced green fluorescent protein (rBPIV3-EGFP) and analyzed fixed cells at 48 h post-infection (hpi). Co-immunostaining for double-stranded RNA (dsRNA), an intermediate in viral RNA synthesis, and the viral N protein revealed colocalization within cytoplasmic inclusion bodies, which is a characteristic feature of viral replication complexes (Fig 1A). When we co-stained with antibodies against m6A and the viral N protein, we observed that m6A exhibited a diffuse distribution in the cytoplasm of mock-infected cells, whereas, in infected cells, m6A distinctly colocalized with the N protein in the punctate dot structures representing inclusion bodies (Fig 1B). These observations were further quantified using Manders’ colocalization coefficients, which confirmed significant colocalization between N protein and dsRNA (Fig 1C) as well as between N protein and m6A (Fig 1D). To further validate these findings, we performed proximity ligation assay (PLA) using antibodies against m6A and N protein, followed by dsRNA immunostaining. PLA signals exhibited the strong colocalization with dsRNA-positive inclusion bodies, and quantitative analysis revealed a significantly higher number of PLA signals in infected cells compared with uninfected controls (S1A and S1B Fig). Moreover, Manders’ colocalization analysis demonstrated robust overlap between PLA signals and dsRNA (S1C Fig).

Given that m6A modification is primarily catalyzed by the methyltransferase METTL3, we investigated its role in viral replication. Overexpression of METTL3 prior to rBPIV3-EGFP infection significantly enhanced viral replication (Fig 1E and 1F). We also assessed the contribution of other m6A regulatory enzymes. Overexpression of the methyltransferase METTL14 similarly enhanced viral replication, whereas overexpression of the demethylases ALKBH5 and FTO showed no significant effect relative to empty vector controls (Fig 1E and 1F). Western blot analysis confirmed that the changes in viral replication correlated with alterations in N protein expression levels (S2A Fig). Conversely, knockdown of METTL3 and METTL14 using a specific shRNA markedly reduced viral replication (Fig 1G and 1H). Western blotting confirmed efficient reduction of endogenous METTL3 and METTL14 levels in shRNA-treated cells (S2B Fig). Furthermore, pretreatment of cells with STM2457, a selective METTL3 inhibitor, significantly attenuated rBPIV3-EGFP replication, without affecting cell viability (Fig 1I, 1J, and 1K). Collectively, these findings demonstrate that m6A modification occurs within viral inclusion bodies, and that METTL3 plays a critical role in BPIV3 replication.

### BPIV3 infection causes recruitment of nuclear METTL3 to cytoplasmic inclusion bodies

**Fig 2:**
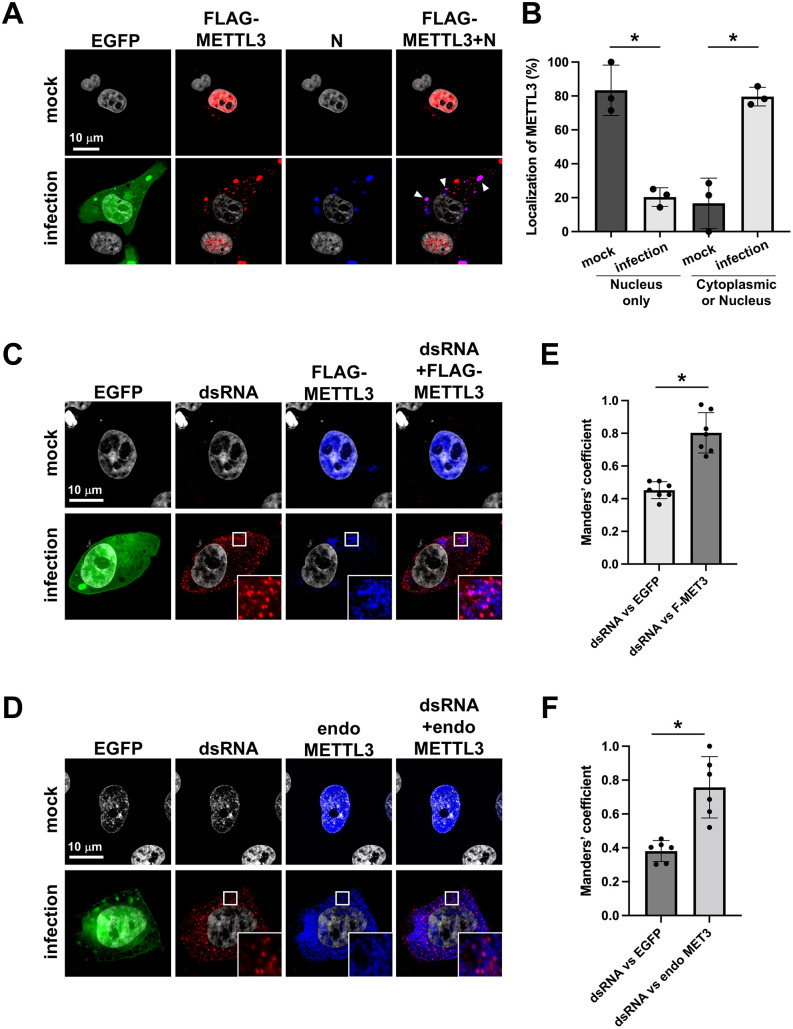
Cytoplasmic translocation of METTL3 during BPIV3 infection and its co-localization with viral dsRNA. HeLa cells were transfected with FLAG-METTL3 expression plasmid, and at 24 h post-transfection (hpt), the cells were infected with rBPIV3-EGFP at an MOI of 1. At 48 h post-infection (hpi), the cells were fixed and costained with anti-FLAG antibody (Ab) for METTL3 and anti-BPIV3-N Ab (A). Arrowheads indicate regions where N and METTL3 fluorescence signals colocalize within the cells. The bar graph shows the distribution of METTL3 subcellular localization. For classification-based quantification, more than 30 METTL3 single- or METTL3/N double-positive cells per condition were randomly selected and classified into two localization patterns (“nucleus only” or “cytoplasmic or nucleus”). The number of cells displaying each localization pattern was counted for each condition from three independent experiments, and the results are presented as the percentage of total cells analyzed (B). HeLa cells were transfected with FLAG-METTL3 plasmid and, 24 hpt, infected with rBPIV3-EGFP at an MOI of 1. At 48 hpi, cells were fixed and co-stained with antibodies against dsRNA and FLAG-METTL3 (C) or dsRNA and endogenous METTL3 (endo METTL3) (D). Representative images from three independent experiments are shown. Manders’ colocalization coefficient between dsRNA and FLAG-METTL3 (E) or dsRNA and endogenous METTL3 (F) fluorescence signals in panels C and D was calculated using the Coloc 2 plugin in Fiji/ImageJ software. The coefficient was determined for each cell, and results represent analyses of 6–7 individual cells per condition from three independent experiments. Bars indicate the mean ± SD. White boxes in panels C and D indicate regions shown as magnified views in the corresponding lower-right insets. Data are representative of three independent experiments (n = 3). Asterisks indicate significance (*p < 0.05); ns, not significant.

METTL3 is predominantly localized in the nucleus, where it catalyzes m6A modification of host mRNA [27] while paramyxovirus RNA synthesis occurs exclusively in cytoplasmic inclusion bodies [20]. For paramyxovirus transcripts to undergo m6A modification, METTL3 must relocate to the cytoplasmic compartment. To test this hypothesis, we transfected the cells with METTL3 and subsequently infected them with BPIV3 to examine METTL3 localization. While METTL3 was exclusively nuclear in mock-infected cells, BPIV3 infection induced a striking translocation of METTL3 to the cytoplasm, where it was specifically recruited to inclusion bodies containing accumulated viral N proteins (Fig 2A). To further demonstrate the reproducibility of these observations, we also acquired low-magnification images (S3 Fig), which provide an overview of multiple A549 cells in the same field and clearly show that METTL3 localization differs between infected and uninfected cells. Quantification of METTL3 distribution confirmed the METTL3 localization shift from the nucleus to the cytoplasm upon infection (Fig 2B). Moreover, cytoplasmic FLAG-METTL3 in infected cells co-localized with dsRNA in the inclusion bodies (Fig 2C), suggesting its direct association with sites of viral RNA synthesis. We further examined the localization pattern of endogenous METTL3 upon BPIV3 infection and found that it predominantly resided within the nucleus in uninfected cells, whereas virus infection redirected endogenous METTL3 to cytoplasmic inclusion bodies (Fig 2D). To quantitatively assess these observations, we calculated Manders’ colocalization coefficient. The analysis demonstrated significant colocalization of FLAG-METTL3 with dsRNA (Fig 2E) as well as endogenous METTL3 with dsRNA (Fig 2F) within the inclusion bodies of infected cells. To provide biochemical support for these imaging data, we performed immunoprecipitation of viral RNA using an anti-dsRNA antibody from UV-crosslinked infected cell lysates. This analysis revealed that both viral N protein and METTL3 were co-precipitated with dsRNA (S4 Fig), further confirming that METTL3 is recruited to viral replication complexes containing dsRNA. This biochemical evidence complements our microscopy findings and strengthens the conclusion that METTL3 is directly associated with viral RNA-containing inclusion bodies. Collectively, these results demonstrate that METTL3 is actively recruited to viral replication complexes where viral transcripts are synthesized, positioning this m6A writer enzyme optimally for modification of nascent viral RNAs.

**Fig 3:**
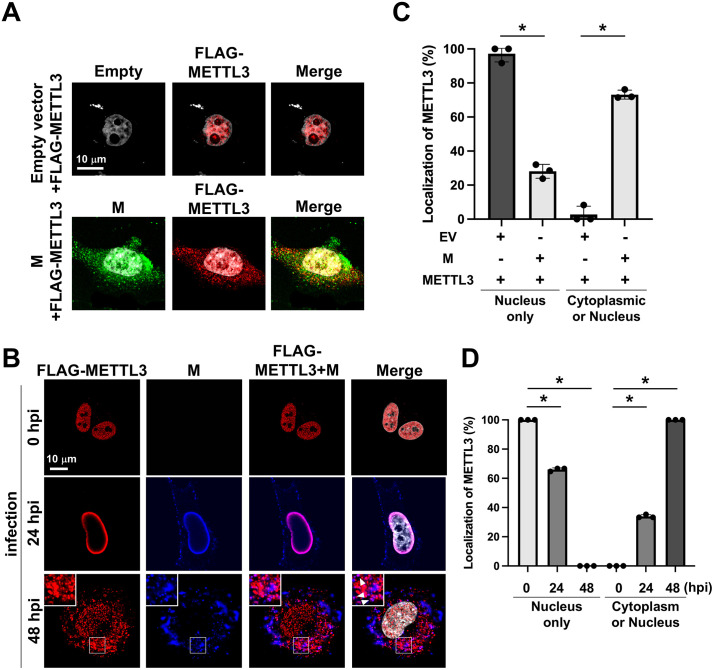
Nucleocytoplasmic translocation of METTL3 during BPIV3 infection and its temporal co-localization with viral M protein. HeLa cells were cotransfected with METTL3 and M expression plasmid or an empty vector. At 48 h post-transfection (hpt), the cells were fixed and costained with anti-BPIV3-M antibody (Ab) and anti-FLAG Ab for METTL3 (A). HeLa cells were transfected with METTL3 expression plasmid, and at 24 hpt, the cells were infected with rBPIV3-EGFP at an MOI of 1. At 48 h post-infection (hpi), the cells were fixed and costained with anti-FLAG antibody (Ab) for METTL3 and anti-BPIV3-M Ab (B). Cell nuclei were stained with DAPI. Insets represent an enlargement of the areas indicated by a small square. Arrowheads indicate cytoplasmic colocalization of M with METTL3. Representative images from three independent experiments are shown. For classification-based quantification, more than 20 METTL3 single-positive or METTL3/M double-positive cells per condition were randomly selected and categorized into two subcellular localization patterns (“nucleus only” or “cytoplasmic or nucleus”). The number of cells in each category was counted, and the data are presented as the percentage of total cells analyzed from n = 3 independent experiments. (C and D). Asterisks indicate statistically significant differences (*p < 0.05); ns, not significant.

### Paramyxovirus matrix protein binds to METTL3 in the nucleus and mediates its translocation to the cytoplasm

The paramyxovirus M protein contains a bipartite nuclear localization signal (NLS) comprising two basic amino acid clusters that interact with importin to facilitate nuclear trafficking. The M protein also possesses a leucine-rich nuclear export signal (NES) that mediates cytoplasmic trafficking [16,28], however, the biological significance of this nucleocytoplasmic shuttling remains elusive. To investigate whether the M protein facilitates METTL3 translocation to the cytoplasm, we co-expressed the M protein and METTL3. We found that the expression of the M protein, but not the N protein, induced a dramatic shift in METTL3 localization from the nucleus to the cytoplasm (Figs 3A and S5). To further assess the temporal dynamics of this process, we co-expressed METTL3 with M protein and collected cells at multiple time points post-transfection. METTL3 was initially retained in the nucleus, but progressively relocalized to the cytoplasm over time, with a substantial cytoplasmic accumulation observed by 48–72 hours post-transfection (S6A and S6B Fig). These results clearly demonstrate that M protein-driven nuclear export of METTL3 is a gradual, time-dependent process.

We further examined the temporal dynamics of subcellular localization of METTL3 and M proteins in BPIV3-infected cells. Immediately after the viral challenge (0 h post-infection; 0 hpi), METTL3 was exclusively localized in the nucleus. By 24 hpi, METTL3 accumulated at the nuclear membrane, colocalizing with M protein. At 48 hpi, both METTL3 and M proteins were translocated to the cytoplasm, where they maintained their colocalization (Fig 3B). Quantitative analysis confirmed that METTL3 shifted from predominantly nuclear to cytoplasmic localization when co-expressed with M protein (Fig 3C) and during the course of viral infection (Fig 3D). These findings strongly suggest that the M protein mediates METTL3 nuclear export to the cytoplasm.

To determine whether the M protein-mediated cytoplasmic translocation of METTL3 is conserved among paramyxoviruses, we tested M proteins from Sendai virus (SeV), human parainfluenza virus type 3 (hPIV3), Nipah virus (NiV), and measles virus (MeV). All the M proteins examined demonstrated the ability to relocalize METTL3 to the cytoplasm (S7 Fig), although co-expression of N and P proteins was required to achieve this effect in hPIV3 and MeV. These results suggest that the ability to translocate METTL3 is a conserved function of the paramyxovirus M proteins.

### Nucleocytoplasmic trafficking of METTL3 is mediated by Exportin-1-dependent nuclear export of paramyxovirus M protein

**Fig 4:**
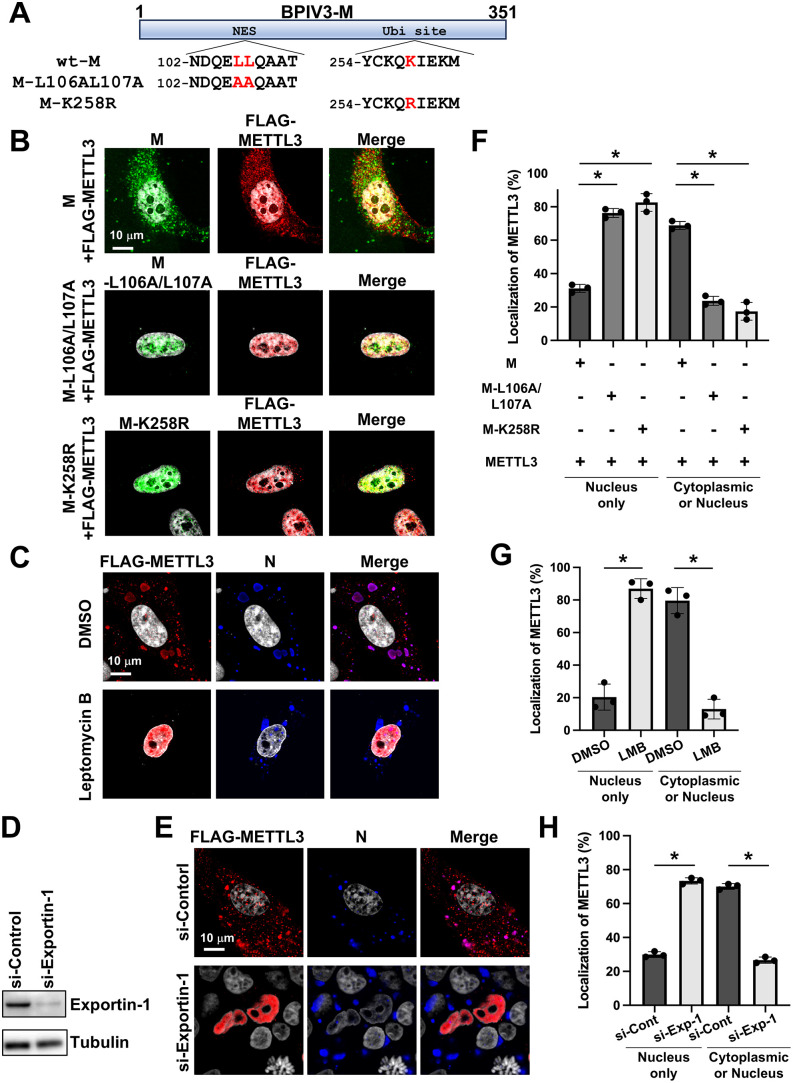
Dependence of METTL3 nuclear export on exportin-1-mediated nuclear export of M protein. (A) Schematic representation of BPIV3-M point mutants. BPIV3-M contains a nuclear export signal (NES) at L106 and L107 and a ubiquitination site (Ubi site) at K258. L106 and L107, or K258 in BPIV3-M, were substituted with alanine (M-L106A/L107A) or arginine (M-K258R) residues as indicated, respectively, and the substituted residue is shown as a red letter. (B) HeLa cells were cotransfected with expression plasmids of wt-BPIV3-M, M-L106A/L107A, and K258R along with FLAG-METTL3 expression plasmid. At 48 h post-transfection (hpt), the cells were costained with anti-M antibody (Ab) and anti-FLAG Ab for METTL3. (C) HeLa cells were transfected with the METTL3 expression plasmid. At 24 hpt, the cells were infected with rBPIV3-EGFP at an MOI of 1. At 4 h post-infection (hpi), DMSO or 5 ng/mL Leptomycin B (LMB) was added, and the cells were incubated for 48 h in the presence of LMB. Then, the cells were fixed, and METTL3 and BPIV3-N were costained. (D) HeLa cells were transfected with siRNA targeting Exportin-1 (si-Exportin-1) or control siRNA (si-Control). At 48 hpt, cells were lysed and subjected to immunoblotting with anti–Exportin-1 antibody to confirm knockdown efficiency. (E) HeLa cells were cotransfected with siRNA for Exportin-1 and METTL3 expression plasmid. At 24 hpt, the cells were infected with rBPIV3-EGFP at an MOI of 1. The cells were fixed at 48 hpi and costained for BPIV3-N and METTL3. Representative images from three independent experiments are shown. For classification-based quantification, more than 20 METTL3/M double-positive cells (F), METTL3/N double-positive cells **(G)**, or METTL3/N double-positive cells under siRNA conditions (H) per condition were randomly selected and categorized into two subcellular localization patterns (“nucleus only” or “cytoplasmic or nucleus”). The number of cells in each category was counted, and the data are presented as the percentage of total cells analyzed from n = 3 independent experiments. Asterisks indicate statistically significant differences (*p < 0.05).

Previous studies demonstrated that substituting two leucine residues with alanine residues in the NES (M-L106A/L107A) inhibits M protein cytoplasmic trafficking, causing nuclear retention [16,28]. These studies also identified lysine 258 (K258) as a ubiquitination site required for nuclear export, with K258R mutation similarly blocking M protein nuclear export [16,28]. To further investigate this nucleocytoplasmic shuttling mechanism, we generated rBPIV3 M-L106A/L107A and M-K258R mutants (Fig 4A) and examined their effect on METTL3 trafficking. As expected, M protein of both rBPIV3 M-L106A/L107A and K258R mutants was retained in the nucleus and failed to facilitate METTL3 translocation to the cytoplasm (Fig 4B).

Exportin-1 is a critical transporter that facilitates cargo protein export from the nucleus to the cytoplasm by binding to NES and mediating transport through nuclear pore complexes [29,30]. To elucidate this mechanism further, we employed two experimental approaches. First, we used Leptomycin B (LMB), which specifically binds to the NES-binding site of exportin-1, thereby disrupting the export of NES-bearing proteins. When cells were pre-treated with LMB, both the M protein and METTL3 failed to translocate to the cytoplasm, even during BPIV3 infection (Fig 4C). Additionally, exportin-1 knockdown using siRNA, confirmed by western blotting (Fig 4D), similarly prevented METTL3 migration from the nucleus to the cytoplasm (Fig 4E). Classification-based quantification further demonstrated that both NES-mutant M proteins (L106A/L107A and K258R) (Fig 4F), LMB treatment (Fig 4G), and exportin-1 knockdown (Fig 4H) significantly reduced cytoplasmic redistribution of METTL3. Collectively, these findings demonstrate that M protein mediates METTL3 translocation through an exportin-1-dependent pathway.

**Fig 5:**
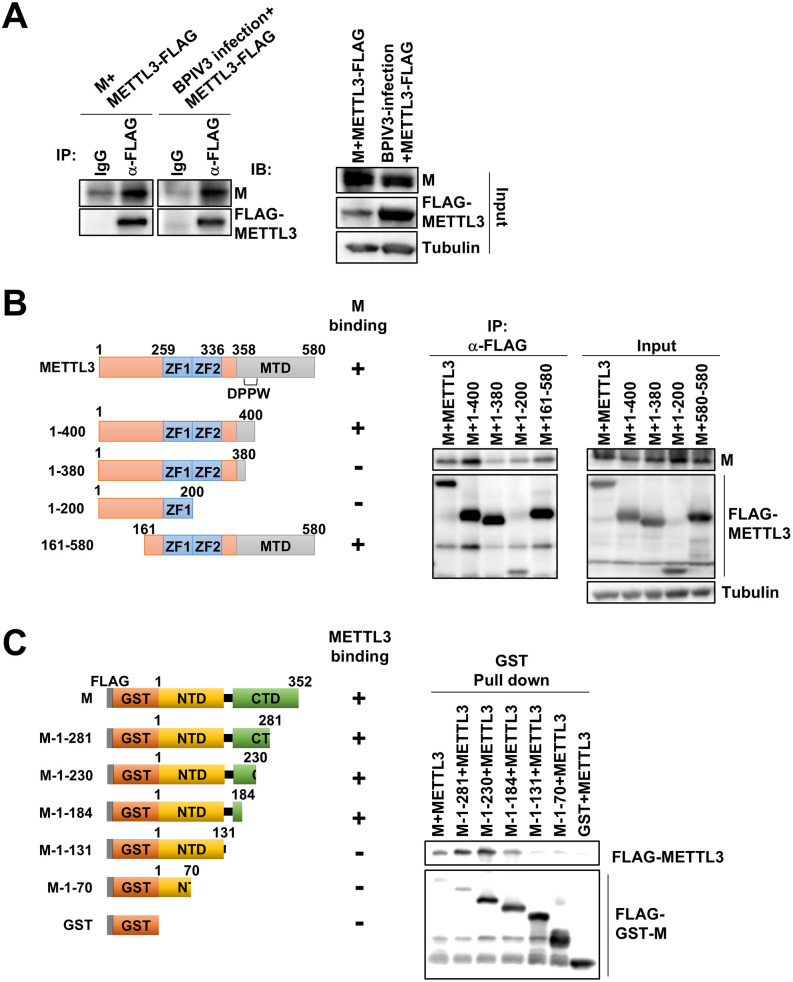
Molecular interaction between M and methyltransferase domain of METTL3. (A) 293T cells were cotransfected with M expression plasmid together with FLAG-METTL3 expression plasmid. For infected cells, the cells were transfected with FLAG-METTL3 expression plasmid and infected with rBPIV3-EGFP at an MOI of 1 at 24 h post-transfection (hpt). At 72 hpt, the cells were harvested and subjected to coimmunoprecipitation with anti-FLAG antibody (Ab). The precipitates were analyzed by western blotting using anti-BPIV3-M Ab. (B) Schematic representation of human METTL3. ZF1 and ZF2: two zinc finger domains, MTD: methyltransferase domain, DPPW: DPPW motif. The 1-400, 1-380, 1-200, and 161-580 deletion mutants lacking each domain are shown. 293T cells were cotransfected with the deletion mutants along with BPIV3-M. At 48 hpt, the cells were harvested and subjected to coimmunoprecipitation with anti-FLAG Ab as described in the legend of Fig 5A. (C) Schematic representation of FLAG- and GST-tagged deletion mutants of the BPIV3 M protein used to map the METTL3 interaction domain. Cells were transfected with a FLAG-METTL3 expression plasmid, and cells were harvested at 48–72 h post-transfection. Deletion mutants of the M protein fused with FLAG and GST tags were synthesized using a wheat germ cell-free expression system. Each FLAG-GST-tagged M deletion mutant was incubated with glutathione–sepharose beads at 4°C, followed by the addition of lysates containing FLAG-METTL3. After incubation, the beads were extensively washed, and bound proteins were analyzed by western blotting. Both METTL3 and M deletion mutants were detected using an anti-FLAG antibody. Each experiment was performed at least twice.

Next, we investigated the molecular interactions between the M protein and METTL3. Co-immunoprecipitation experiments confirmed robust binding between the M protein and METTL3 (Fig 5A). METTL3 comprises two zinc finger domains (ZF1 and ZF2) and a methyltransferase domain (MTD) [31,32]. To precisely map the interaction regions, we generated domain-deletion mutants (Fig 5B). Co-immunoprecipitation experiments with these mutants revealed that the M protein binds to a region spanning amino acids 381–400 within the methyltransferase domain, which includes the catalytically critical DPPW motif. This was particularly evident from the significantly reduced interaction observed with the 1–380 mutant lacking the DPPW motif (Fig 5B).

Next, we examined the binding region of the viral M protein to METTL3. Since M protein deletion mutants are unstable and difficult to express in living cells, we fused a series of M deletion mutants to a GST tag and expressed them in a wheat cell-free protein production system (Fig 5C). Subsequent GST pull-down analysis revealed that M protein binds to METTL3 via an amino acids 132–184 region (Fig 5C). These results indicate that the central domain of viral M protein interacts with METTL3-MTD.

### METTL3 catalyzes m6A modification and enhances BPIV3-N mRNA stability

METTL3 recognizes a specific RNA sequence motif (RRACH, where R = A/G and H = U/A/C) in the substrate RNAs for m6A modification. The S-adenosyl methionine (SAM)-binding domain of METTL3 serves as a methyl group donor to add a methyl group to adenosine within this consensus motif [32]. To predict the epitranscriptomic profile of BPIV3, we employed a Sequence-based RNA Adenosine Methylation site Predictor (SRAMP) algorithm, which predicts potential m6A modification sites. Our SRAMP analysis revealed high m6A probability scores in multiple regions, with particularly strong signals in both the N and P open reading frames (S8 Fig). Since the N protein is an essential structural component for nucleocapsid formation and is the most abundantly expressed viral protein during infection, we focused our subsequent analyses on the N gene transcripts as potential targets for m6A modification.

**Fig 6:**
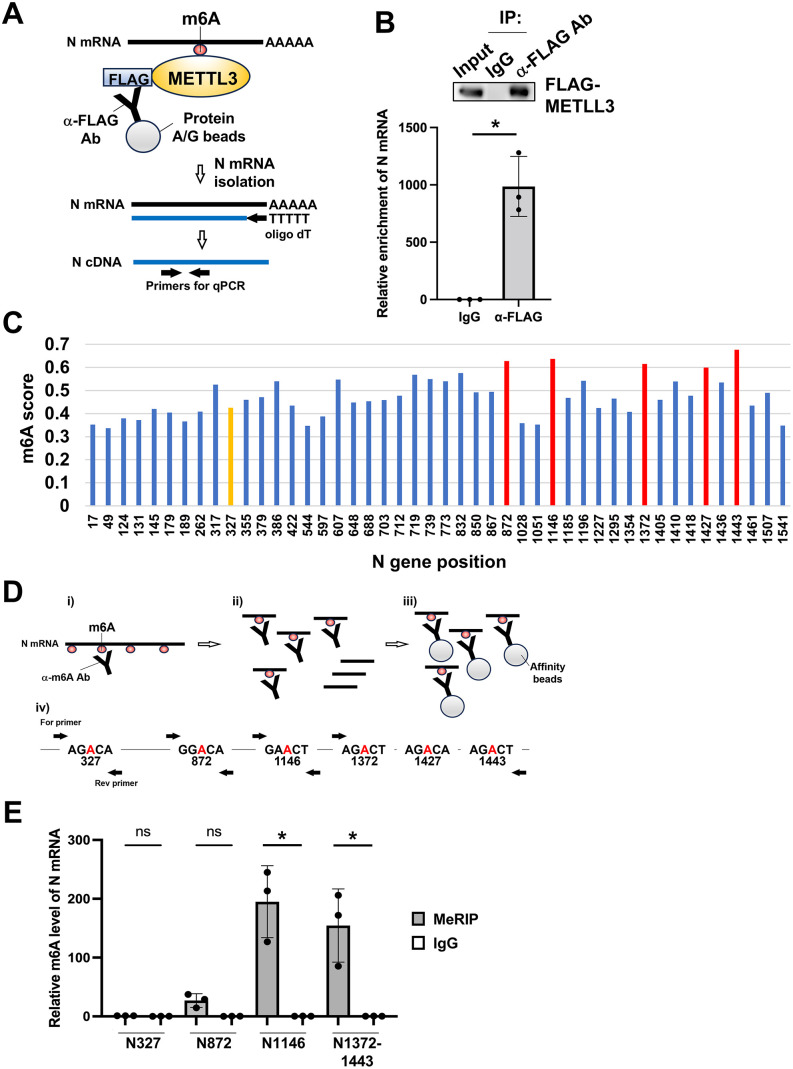
Binding of METTL3 to BPIV3-N mRNA and m6A modification analysis of N mRNA at SRAMP-predicted sites. (A) A schematic diagram of the RNA immunoprecipitation (RIP) assay used to detect METTL3-associated N mRNA. (B) 293T cells were transfected with METTL3 expression plasmids. At 24 h post-transfection (hpt), the cells were infected with rBPIV3-EGFP at an MOI of 1 for 48 **h.** The infected cells were lysed, and the extracts were subjected to RNA immunoprecipitation assay with anti-FLAG antibody (Ab). After immunoprecipitation, cDNA of BPIV3-N mRNA captured by METTL3 was synthesized and the amount of N mRNA was quantified by qPCR. Followed by normalization to input RNA values, relative enrichment values of N mRNA were expressed relative to control IgG values using the ΔΔCt method. (C) SRAMP was used to predict the m6A site of the N gene derived from the BPIV3 genome. The vertical axis shows the m6A score and the horizontal axis shows the position number of the base of the N gene where N6-methyladenosine is present within the m6A motif. A yellow bar indicates low-scoring m6A site, N327, and red bars indicate high-scoring m6A sites, N872, N1146, N1372, N1427, and N1443. (D) Schematic representation of the MeRIP (m6A RNA immunoprecipitation) assay. (i) The m6A-containing RNA fragments were bound by an m6A-specific Ab. (ii) Viral N mRNA containing m6A modifications was fragmented. (iii) Antibody–RNA complexes were captured using protein affinity beads. (iv) RNA was eluted, reverse transcribed into cDNA, and amplified by qPCR using primers specific for the indicated N gene regions (N327, N872, N1146, and N1372–1443). (E) 293T cells were infected with rBPIV3-EGFP at an MOI of 1. At 72 h post-infection, the infected cells were harvested, and only mRNA from total RNA was purified. The m6A-modified mRNA was immunoprecipitated with m6A-specific Ab and magnetic beads. Followed by cleavage of the captured mRNA with the cleavage enzyme, the m6A-specific Ab-captured mRNA was eluted from magnetic beads and purified. cDNA was synthesized by reverse transcription reaction, and the m6A sites in N mRNA were then identified by qPCR. The relative value of each to the mRNA value of input RNA was calculated using the ΔΔCt method. Data represent the mean ± SD from n = 3 independent experiments. Asterisks represent statistically significant differences (* *p* < 0.05). ns; not significant.

To determine whether the N gene is indeed regulated by METTL3-mediated epitranscriptomic modification, we first examined whether METTL3 physically interacts with BPIV3 N mRNA. We performed RNA immunoprecipitation (RIP) assays using cell lysates collected from FLAG-tagged METTL3-expressing cells infected with BPIV3. Following immunoprecipitation with anti-FLAG antibody, the amount of N mRNA bound to METTL3 was quantified by RT-PCR (Fig 6A). Compared to immunoprecipitates with control IgG, N mRNA substantially co-precipitated with METTL3 (Fig 6B), indicating a specific interaction between METTL3 and viral N transcripts.

Next, we mapped precise m6A sites within the N gene ORF using methylated RNA immunoprecipitation followed by quantitative reverse transcription PCR (MeRIP-qRT-PCR). Total RNA from BPIV3-infected cells was chemically fragmented into ≤ 100-nucleotide fragments and subjected to immunoprecipitation with a monoclonal antibody against m6A. The immunoprecipitated RNA fragments were analyzed by qRT-PCR using primer sets flanking potential m6A modification sites with high SRAMP scores: N872, N1146, N1372, N1427, and N1443 in BPIV3 N mRNA (Fig 6C and 6D). Our analysis revealed significant m6A enrichment in regions containing N872, N1146, and N1372-1443, whereas N327, which had a low SRAMP score, showed minimal precipitation with the m6A antibody (Fig 6E). These results provide experimental evidence that the computationally predicted m6A sites within N gene transcripts are indeed catalytically modified.

**Fig 7:**
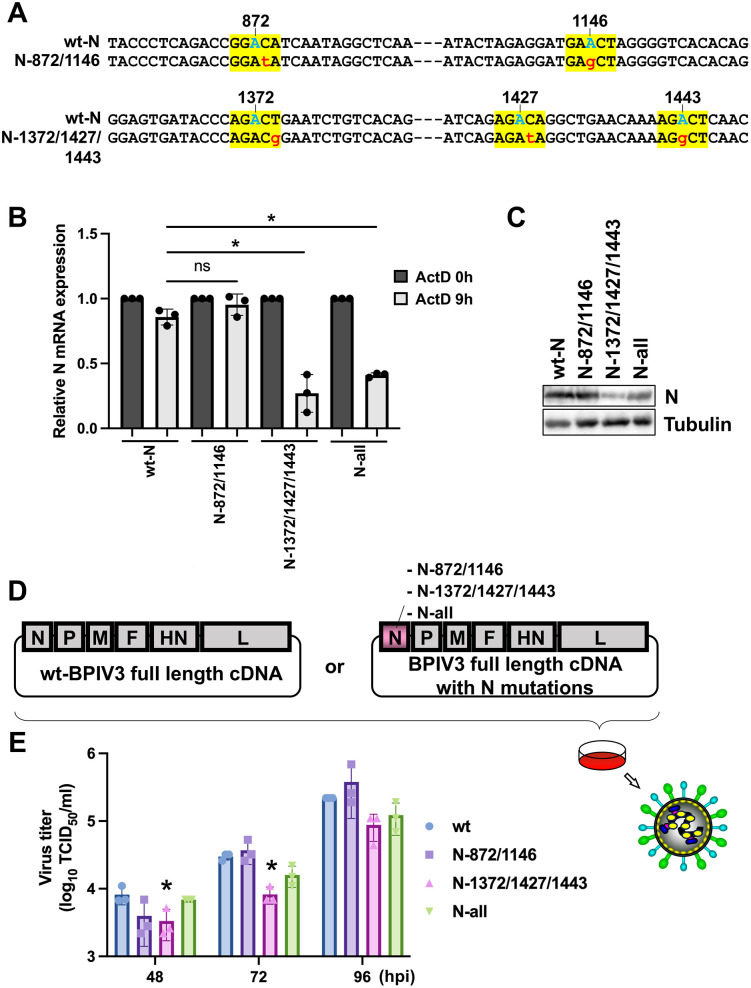
Role of N mRNA m6A modifications in RNA stability and efficient viral replication. (A) Schematic representation of the N gene with mutations to substitute the critical A, C, or T in the m6A motif. The m6A motif, the putative N6 adenosine, and the mutation sites are indicated by yellow squares, cyan, and red characters, respectively. (B) 293T cells were transfected with wt-N, N-872-1146, N1372-1443, and N-all mutants expression plasmids. At 48 h post-transfection (hpt), the cells were treated with 5 μg/mL ActD for 9 h. After drug treatment, the total RNA was extracted, and cDNA of N mRNA was synthesized by reverse transcription reaction. The amount of N mRNA was quantified by qRT-PCR. For quantification, followed by normalization for the N mRNA values by the β-actin values, the stability of each N mRNA was expressed as a value relative to that of 0 h of ActD treatment. (C) 293T cells were transfected with wt-N, N-872-1146, N1372-1443, and N-all mutants expression plasmids. At 48 hpt, the cells were harvested and subjected to western blotting using anti-BPIV3-N Ab (n = 2). (D) Schematic representation of recombinant BPIV3 genome constructs used in this study. The wt BPIV3 full-length cDNA contains the N, P, M, F, HN, and L genes in the native order. The mutant BPIV3 full-length cDNA carries specific point mutations in the N gene (N-872/1146, N-1372/1427/1443, or N-all), indicated in pink. These constructs were used to generate recombinant viruses for subsequent infection experiments in cell culture. (E) Cells were infected with the wt or m6A mutant viruses at an MOI of 1. At various time points (48, 72, and 96 hpi), the culture supernatant was harvested, and the virus titers were measured by TCID_50_ assay on MDBK cells. Data represent the mean ± SD from n = 3 independent experiments. Asterisks represent statistically significant differences (* *p* < 0.05). ns; not significant. Schematic illustration in panel D was created with MS PowerPoint.

To assess the functional significance of these m6A modifications, we introduced point mutations to disrupt m6A consensus sequences in the N gene while maintaining amino acid coding through synonymous substitutions. We constructed three non-m6A mutants: one harboring mutations at positions N872 and N1146 (N-872/1146); another with mutations at positions N1372, N1427, and N1443 (N-1372/1427/1443); and a third containing mutations at all five m6A sites (N-all) (Fig 7A). These constructs were transfected into 293T cells and analyzed for mRNA decay kinetics to determine the transcript stability following transcriptional inhibition with actinomycin D (ActD). Our results demonstrated that both the N-1372/1427/1443 and N-all mutants exhibited significantly reduced mRNA stability compared to the wild-type (wt) N transcripts. Interestingly, the N-872/1146 mutant showed stability comparable to that of wt-N (Fig 7B), suggesting that m6A modifications at positions 1372, 1427, and 1443 play a critical role in maintaining N transcript stability. These differential effects on mRNA stability were also reflected at the protein level, as assessed by western blotting (Fig 7C), with reduced N protein expression observed in mutants with decreased mRNA stability.

To further validate these findings, we performed a minigenome assay using either wild-type N (wt-N) or N-1372/1427/1443 mutant expression plasmid (S9A Fig). Consistent with the reduced protein levels observed in Fig 7C, NanoLuc activity in the minigenome system was markedly decreased in the N-1372/1427/1443 mutant compared to wt-N, indicating impaired support for viral RNA synthesis.

Finally, to determine the impact of these m6A modifications on viral replication, we generated recombinant BPIV3 viruses harboring the same mutations (N-872/1146, N-1372/1427/1443, and N-all) within the viral genome sequence (Fig 7D). Consistent with our mRNA stability findings, replication of N-1372/1427/1443 mutant virus was significantly attenuated compared to that of the wt virus at 72 hpi (Fig 7E). To investigate whether the reduced viral replication observed with the N-1372/1427/1443 mutant resulted from decreased viral protein expression, we analyzed N and M protein levels in A549 and HeLa cells infected with either wild-type or N-1372/1427/1443 mutant viruses. Western blot analysis revealed comparable M protein levels between wild-type and mutant infections, whereas N protein expression was markedly reduced in cells infected with the N-1372/1427/1443 mutant (S9B Fig). These findings suggest that the impaired replication capacity of the mutant virus is closely linked to diminished N protein expression.

To test whether reduced N expression was responsible for the decreased viral titers, we performed a rescue experiment in which 293T cells were transfected with either an N expression plasmid or an empty vector prior to infection with wt or N-1372/1427/1443 mutant virus. Exogenous N supply significantly restored viral replication of the mutant virus compared to the empty vector control (S9C Fig). To investigate the molecular basis of the rescue effect, we extracted total RNA from infected cells and performed strand-specific RT-PCR followed by qPCR to quantify viral genomic and antigenomic RNA. Consistent with the observed recovery of viral titers, exogenous N protein expression significantly restored the synthesis of both viral genome (S9D Fig) and antigenome (S9E Fig) in cells infected with the N-1372/1427/1443 mutant virus compared to the empty vector control. However, neither the viral titers nor the genome/antigenome RNA levels fully returned to wild-type levels, even with exogenous N supplementation. This suggests that loss of the m6A sites within the N gene region of the genome/antigenome may also directly impair the stability of viral RNAs, in addition to reducing N protein expression.

### Matrix protein attenuated host immune response by altering the m6A modification of interferon-β mRNA

While our above data demonstrated that the viral M protein facilitates m6A modification of viral transcripts by recruiting METTL3 to cytoplasmic replication complexes, we hypothesized that this relocalization might simultaneously deplete nuclear METTL3, thereby disrupting m6A modification of host mRNAs, particularly those involved in innate immune responses. To investigate the transcriptome-wide effects of M protein-induced nuclear METTL3 depletion, we transfected A549 cells with either M protein expression plasmids or empty vectors, stimulated them with polyinosinic-polycytidylic acid (poly(I:C)) to mimic viral infection, and performed RNA-sequencing (RNA-seq) analysis.

**Fig 8:**
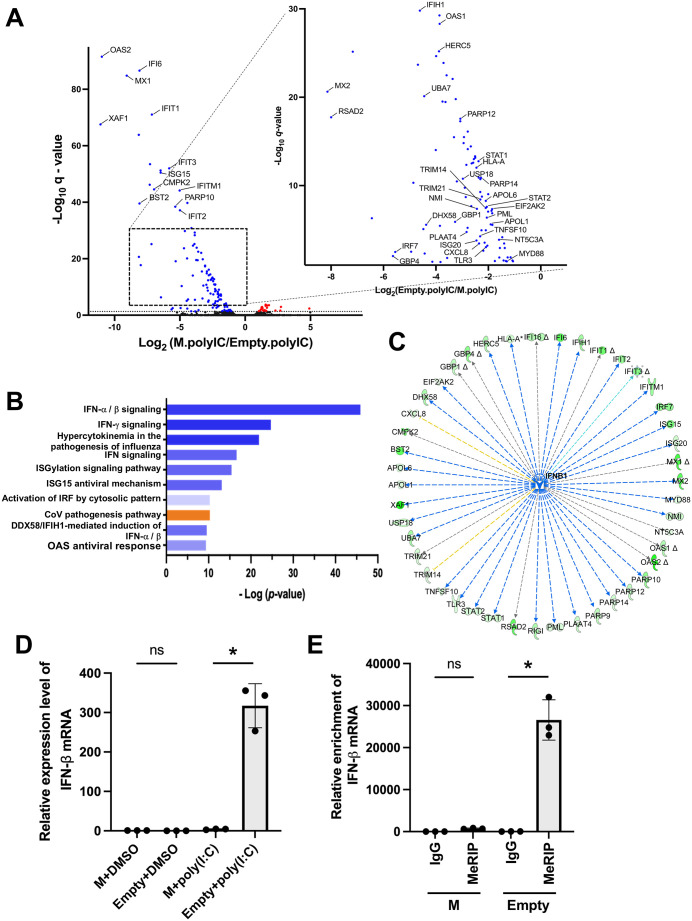
Reduction of m6A modification and mRNA expression of type I interferon genes by M expression. A549 cells were transfected with an M expression plasmid or an empty vector and treated with 10 μg/ml poly(I:C) for 24 h. Total RNA was extracted and subjected to RNA sequencing. (A) Volcano plots illustrating differential gene expressions between cells transfected with the M protein expression plasmid (M.poly(I:C)) and those transfected with the empty vector (Empty.poly(I:C)). The x-axis represents log_2_ fold changes, while the y-axis represents -log_10_ adjusted *p*-values (q-values). Red and blue plots represent genes upregulated and downregulated by M expression, respectively. The inset zooms in on genes with moderate fold changes and highlights downregulated genes, with an enlarged view shown on the right. Genes with significant differential expression, especially under the control of IFN-β, are labeled. (B) Top 10 canonical pathways determined by IPA. The orange or blue nodes indicate predicted biological function activation or inhibition, respectively. The x-axis indicates the -log_10_ p-values for each pathway, with the most significant terms related to interferon signaling. (C) IPA network depicting the pathways and gene interactions. Green nodes represent DEGs, and the edges indicate known interactions. Solid lines and dashed lines represent direct and indirect interactions, respectively. The degree of expression and prediction is reflected by the intensity of the color. (D) Total RNA was extracted from cells transfected with either M expression plasmid or empty vector after stimulation with poly(I:C) or DMSO addition, as prepared in A. The amount of IFN-β mRNA was quantified by qPCR using IFN-β gene-specific primer sets. (E) 293T cells were cotransfected with IFN-β + 3’UTR expression plasmid together with either M expression plasmid or empty vector. Total RNA extracted from the cells was subjected to m6A RNA immunoprecipitation using an m6A-specific antibody. IFN-β m6A modification efficiency was calculated by qPCR using primer sets designed to detect m6A sites within IFN-β mRNA, with enriched m6A-modified RNA as a template. Data represent the mean ± SD from n = 3 independent experiments (except for RNA-seq, which was performed once as a screening experiment). Asterisks represent statistically significant differences (* *p* < 0.05). ns; not significant.

RNA-seq results revealed significant transcriptional alterations in response to M protein expression in poly(I:C)-stimulated A549 cells. Volcano plot analysis demonstrated a pronounced pattern of transcriptional suppression, with numerous genes significantly downregulated (blue dots) and relatively few genes upregulated (red dots) compared to the empty vector control (Fig 8A). This asymmetric distribution of differentially expressed genes suggested a broad suppressive effect of M protein on host gene expression.

Gene Ontology analysis of the differentially expressed genes revealed strong enrichment in interferon signaling and antiviral response pathways. The most significantly enriched functional terms were predominantly associated with interferon-mediated cellular responses (Fig 8B), indicating that M protein expression specifically impacts host antiviral defense mechanisms. To further characterize the regulatory relationships among these differentially expressed genes, we performed a pathway interaction network analysis. This analysis identified interferon-β (IFN-β) as a critical upstream regulator orchestrating the transcriptional response.

The network diagram revealed a hierarchical structure with IFN-β occupying a central node position and connecting multiple downstream effector genes involved in antiviral and immune responses (Fig 8C). This network architecture suggests that IFN-β functions as a master regulator of the transcriptional changes induced by M protein expression, controlling various aspects of the cellular immune response through its downstream targets. Given the central role of IFN-β in this regulatory network, we specifically quantified IFN-β mRNA levels in cells expressing M protein and subsequently stimulated them with poly(I:C). Consistent with our RNA-seq findings, qPCR analysis demonstrated that IFN-β mRNA expression was significantly reduced in M protein-expressing cells compared to that in the empty vector-transfected controls (Fig 8D).

To elucidate the mechanistic basis for this reduction, we examined the m6A modification status at two major m6A sites within the IFN-β gene, one located near the 3’ end of the coding sequence and the other in the 3’ untranslated region [33], using methylated RNA immunoprecipitation followed by qPCR analysis. Remarkably, the efficiency of m6A modification at both sites was substantially diminished in M protein-expressing cells (Fig 8E), which correlated with the reduced IFN-β expression observed earlier.

To further clarify the role of METTL3 in regulating IFN-β production, we first examined whether the M-K258R mutation affects the subcellular localization of METTL3. In cells infected with wild-type rBPIV3, both the M protein and METTL3 translocated from the nucleus to the cytoplasm at 48 hpi, where they colocalized. In contrast, infection with the M-K258R mutant virus failed to induce cytoplasmic translocation, and METTL3 remained restricted to the nucleus together with M-K258R (S10A Fig). Quantitative analysis confirmed this observation, demonstrating that cytoplasmic relocalization of METTL3 was abolished in M-K258R-infected cells (S10B Fig).

We next investigated the functional consequences of this altered localization on IFN-β expression. qPCR analysis revealed that IFN-β mRNA levels were significantly higher in cells infected with the M-K258R mutant compared to wild-type rBPIV3 (S10C Fig). To assess whether this increase was linked to changes in RNA modification, we performed MeRIP assays using an m6A-specific antibody. IFN-β transcripts from M-K258R-infected cells exhibited substantially higher m6A modification efficiency than those from wild-type-infected cells (S10D Fig).

Finally, to determine whether METTL3 expression itself directly influences IFN-β production, we compared METTL3-overexpressing and METTL3-knockdown cells. BPIV3 infection of METTL3-overexpressing cells led to markedly elevated IFN-β mRNA levels compared to empty vector controls (S10E Fig). Conversely, stable METTL3 knockdown significantly reduced IFN-β expression relative to control shRNA cells (S10F Fig).

Collectively, these data suggest a viral evasion strategy whereby M protein induces nuclear METTL3 depletion, resulting in impaired m6A modification of IFN-β mRNA and attenuation of the host anti-viral response.

## Discussion

**Fig 9:**
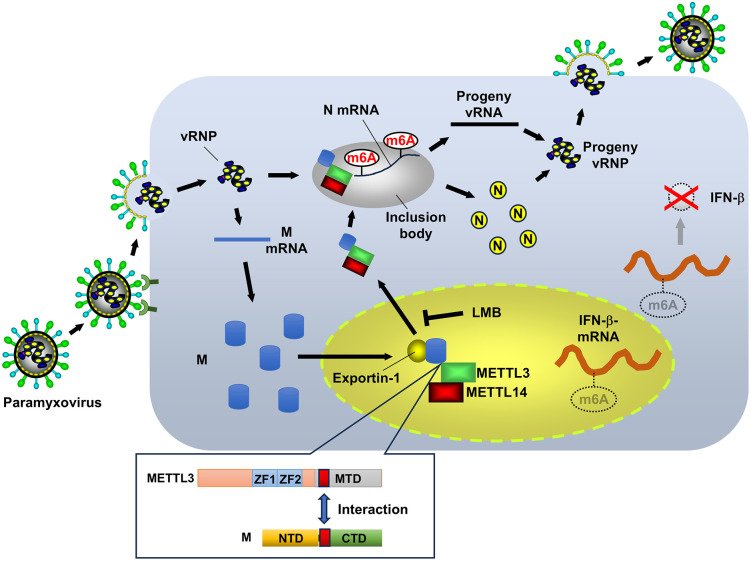
A model for m6A modification of viral and host RNA during paramyxovirus infection. The newly synthesized paramyxovirus matrix (M) protein enters the nucleus and binds to METTL3 and METTL14 methyltransferase complexes. Exportin-1 recognizes M protein, facilitating the export of M-writer complexes to the cytoplasm. These complexes are then recruited into cytoplasmic inclusion bodies, which serve as viral RNA synthesis sites. Within these structures, m6A writers modify viral RNA, particularly N mRNA. The nuclear efflux of METTL3, driven by M protein, depletes nuclear m6A writers, leading to abnormal m6A modification of host mRNAs. Interferon-beta (IFN-β) mRNA is especially affected, showing reduced m6A levels. Consequently, reduced IFN-β mRNA levels result in attenuated antiviral and immune responses, allowing for more efficient viral replication. This process represents a sophisticated viral strategy to enhance replication while simultaneously suppressing host defense mechanisms, illustrating how paramyxoviruses manipulate cellular m6A machinery to their advantage. Schematic illustration in this Fig was created with MS PowerPoint.

The cytoplasmic nature of paramyxovirus replication presents a unique challenge as these viruses have evolved sophisticated mechanisms to optimize their replication strategy within the cytoplasmic compartment. Our study reveals that the paramyxovirus matrix protein plays a critical role in translocating the m6A methyltransferase METTL3 from its normal nuclear location to viral cytoplasmic replication factories. This strategic relocalization serves a dual purpose; it enhances viral gene expression by increasing m6A modifications on viral transcripts, while simultaneously depleting nuclear METTL3, thereby reducing m6A modifications on host antiviral transcripts (Fig 9). This coordinated epitranscriptomic modulation represents a previously unrecognized mechanism by which paramyxoviruses simultaneously enhance their replicative fitness and evade host immunity, a viral adaptation strategy that maximizes the utility of a single host factor for multiple aspects of the viral life cycle.

Our understanding of m6A biology has advanced, and so has our recognition of its critical role in viral infection. Recent studies have demonstrated that m6A functions in the cytoplasm during viral replication, as observed in SARS-CoV-2 [26,34,35] and vesicular stomatitis virus infections [36,37], however, the detailed molecular mechanisms remain poorly characterized. One of the most striking findings of our study is that the paramyxovirus M protein promotes nuclear export of METTL3, which in turn impairs host antiviral defense by reducing m6A modification of IFN-β transcripts. Consequently, IFN-β expression is markedly suppressed during wild-type virus infection, whereas the export-deficient M-K258R mutant fails to deplete nuclear METTL3, leading to preserved m6A modification and elevated IFN-β expression (S10 Fig). This provides direct mechanistic evidence that paramyxoviruses suppress innate immunity by redistributing METTL3 away from the nucleus, thereby dampening interferon responses and facilitating immune evasion.

Our current study provides novel insights into how paramyxoviruses, whose replication occurs entirely within the cytoplasm, manipulate host epitranscriptomic machinery to promote their replication cycle. Through reverse genetics, we confirmed the functional significance of m6A modifications of viral N mRNA, as mutations in the m6A acceptor sites significantly attenuated viral replication capacity. Importantly, our findings suggest that the functional significance of m6A modification extends beyond regulating the stability of a specific viral mRNA such as N, but may also encompass viral genomic and antigenomic RNAs (S9 Fig). While the reduced replication of the N-m6A mutants can be largely attributed to insufficient N protein production, the incomplete rescue of viral titers and RNA synthesis by exogenous N expression implies that loss of m6A sites within the N gene region may additionally compromise the stability or processing of genome/antigenome RNA. This highlights the possibility that m6A marks exert broader effects on multiple layers of the viral replication cycle beyond individual transcript regulation. Future studies will be required to directly assess whether the loss of m6A modification within the N gene region affects the stability and turnover of genome and antigenome RNAs themselves, which may represent an additional layer of regulation contributing to the attenuated phenotype.

Our findings establish a direct link between specific epitranscriptomic marks and viral fitness. Moreover, our data demonstrate that this epitranscriptomic dysregulation simultaneously attenuates host antiviral responses, creating a more favorable environment for viral replication. Comprehensive transcriptome and Gene Ontology analyses revealed that the paramyxovirus M protein specifically downregulates genes related to interferon signaling and antiviral response pathways in poly(I:C)-stimulated cells. Further investigation of the m6A modification status of the IFN-β gene showed that m6A modifications at two major acceptor sites were substantially diminished in M protein-expressing cells. This reduction in m6A marks correlated with decreased IFN-β expression, suggesting a mechanism for immune evasion. Consistent with this model, experiments using the M-K258R mutant virus, which fails to export METTL3, demonstrated impaired cytoplasmic relocalization of METTL3 and resulted in significantly higher IFN-β expression and increased m6A modification efficiency of IFN-β transcripts. Our findings thus uncover a novel mechanism by which paramyxoviruses manipulate host epitranscriptomic regulation to simultaneously enhance viral gene expression and suppress host innate immunity that could be targeted for therapeutic intervention.

Using BPIV3 as a model paramyxovirus, we uncovered a unique viral strategy to hijack METTL3, a predominant nuclear protein, for cytoplasmic functions. Unlike nuclear-replicating viruses such as HIV-1 and influenza A viruses, which utilize METTL3 in their native nuclear environment [38–40], paramyxoviruses employ their M proteins to directly interact with METTL3 and facilitate its export to the cytoplasm through an exportin-1-dependent pathway (Fig 4). This function depends on both the NES domain (L106/L107) and a ubiquitination site (K258), as both mutations abrogated nuclear export of M protein and consequently blocked METTL3 relocalization (Fig 4B). This mechanism represents a previously unrecognized adaptation that enables cytoplasmic RNA viruses to access and exploit the nuclear epitranscriptomic machinery.

The paramyxovirus M protein is a multifunctional structural protein that not only constitutes the viral particle but also traffics to multiple subcellular compartments, where it exerts diverse physiological effects [14,41,42]. M proteins are known to bind to transcription factors and modulate host gene expression, and they have been implicated in host ribosome synthesis through nucleolar localization [43,44]. Collectively, these functions alter the intracellular environment to promote viral replication. Our study adds to this repertoire by demonstrating that paramyxovirus M-proteins can significantly reshape host mRNA m6A patterns by translocating METTL3 to the cytoplasm, thereby modulating gene expression, particularly that of genes associated with innate immune responses (Fig 8). This epitranscriptomic regulation of host gene expression by M proteins may provide critical insights into the multifaceted strategies employed by paramyxoviruses.

Our findings fundamentally reshape our understanding of how cytoplasmic RNA viruses regulate gene expression. The paramyxovirus replication complex has emerged as a critical site for m6A modification, revealing a novel aspect of virus-host interactions. Conservation of this mechanism across multiple paramyxovirus species, including hPIV3, SeV, MeV, and NiV, underscores its evolutionary significance (S7 Fig). Notably, the NES encoded in the N-terminus of the M proteins of various paramyxoviruses ensures this function [16,28], and the conservation of these regions throughout molecular evolution suggests strong negative selection, highlighting its biological importance.

The significance of m6A in viral infections extends beyond paramyxoviruses, with context-dependent effects across different viral families. While m6A marks on the 5’-untranslated region of SARS-CoV-2 genomic RNA promote cap-independent translation during cellular stress [45], m6A modifications of HBV transcripts suppress viral protein expression, potentially contributing to viral persistence [46]. These contrasting outcomes highlight the complexity of m6A-mediated regulation in virus-host interactions. Additionally, many viruses actively manipulate the cellular m6A machinery through protein-protein interactions. The Zika virus RNA contains abundant m6A modifications regulated by host enzymes, which affect viral replication and alter host mRNA methylation patterns [47,48], whereas the hepatitis C virus core protein interacts with m6A reader proteins YTHDF, possibly counteracting their antiviral effects [25]. Our identification of the specific interaction between the paramyxovirus M protein and METTL3 adds to this emerging pattern of viral epitranscriptomic manipulation.

Therapeutically, these findings suggest that small molecules designed to disrupt the M protein-METTL3 interaction could provide a specific antiviral strategy without compromising normal cellular functions. By targeting this virus-specific interaction rather than the broader m6A machinery, such interventions might offer selective inhibition of viral replication while minimizing effects on host cell processes. In this study, we experimentally demonstrated that the central region of the M protein (amino acids 132–184) directly binds to the methyltransferase domain (MTD) of METTL3 (Fig 5), providing a defined molecular interface that could be exploited for therapeutic targeting. Several approaches could be envisioned, including peptide mimetics that compete for the binding site we identified in the methyltransferase domain of METTL3, or small molecules that induce conformational changes in either protein to prevent complex formation. However, given the fundamental role of m6A in cellular processes, potential off-target effects must be carefully considered [49]. The m6A epitranscriptomic landscape is intimately involved in numerous aspects of RNA metabolism, including splicing, export, stability, and translation efficiency of many cellular transcripts. Even targeted interventions might disrupt the delicate balance of these processes in uninfected cells. Additionally, the conservation of the M protein-METTL3 interaction across paramyxoviruses, while therapeutically promising for broad-spectrum applications, suggests this interface may involve structurally conserved domains that share features with host protein interactions.

In conclusion, our study reveals a sophisticated mechanism by which paramyxoviruses manipulate the host m6A machinery to enhance viral replication while suppressing immune responses. The viral M protein serves as a critical mediator in this process, facilitating cytoplasmic relocalization of METTL3 and the subsequent reprogramming of both viral and host epitranscriptomes. This work not only advances our understanding of virus-host interactions but also identifies potential targets for novel antiviral strategies. Future research in this area promises to yield innovative therapeutic approaches while illuminating broader questions of host-pathogen co-evolution and epitranscriptomic regulation in biological systems.

## Materials and methods

### Ethics statement

The human cell lines used in this study include commercially available resources from ATCC and RIKEN BRC. All experiments involving recombinant DNA were approved by the National Institute of Infectious Disease Committee (approval No. 2880) and the Ministry of Education, Culture, Sports, Science and Technology in Japan (approval no. 46 on April 9th, 2024) for Experiments using Recombinant DNA and Pathogens.

### Cells and viruses

293T (ATCC: CRL-11268), HeLa (ATCC: CCL-2), A549 (ATCC: CCL-185), BHK/T7-9 (RIKEN BRC: RCB4942), and MDBK cells were maintained in Dulbecco’s modified Eagle medium supplemented with 10% fetal bovine serum. The MDBK cells were obtained from a collaborator; however, the original source was unclear. METTL3- and METTL14-knockdown (KD) HeLa cells were established by transfection of the gene-specific shRNA expression plasmids for METTL3 (Santa Cruz, Cat. #sc-92172-S) and METTL14 (Santa Cruz, Cat. # sc-89054-SH), and then selected with 1.5 μg/ml puromycin (InvivoGen, Cat. #ant-pr-1). Recombinant BPIV3 derived from the complete sequence of the BN-1 strain and expressing EGFP (rBPIV3-EGFP) was generated by the reverse genetics method in a previous report in our laboratory and used as a wt virus [50]

### Antibodies and reagents

Rabbit anti-BPIV3-N and M polyclonal antibodies (pAbs) were generated as previously described [42]. The following antibodies were used as primary Abs: mouse anti-MeV-M monoclonal Ab (mAb) (clone E388) kindly provided by T. A. Sato, mouse anti-FLAG Ab (proteintech, Cat. #66008–7-Ig), rabbit-FLAG Ab (proteintech, Cat. #66008–4-Ig), mouse anti-HA monoclonal Ab (mAb) (proteintech, Cat. #66006–2-Ig), rabbit anti-HA tag pAb (MBL, Cat. #561), rabbit anti-CRM1 (exportin-1) pAb (proteintech, Cat. #27917–1-AP), mouse anti-double-stranded RNA Ab clone J2 (Nordic MUbio, Cat. #10010200), mouse anti-m6A mAb (Sigma, Cat. #MS1124), rabbit anti-METTL3 pAb (GeneTex, Cat. #GTX105037), rabbit anti-METTL14 pAb (Sigma-Aldrich, Cat. #HPA038002), and HRP-conjugated alpha tubulin mAb (proteintech, Cat. #HRP-66031). LMB, ActD, STM2457, and poly (I:C) were purchased from Sigma-Aldrich (Cat. #L2913), Fuji Film (Cat. #018–21264), MedChemExpress (Cat. #HY-134836), and Novus Biologicals (Cat. #NBP2–25288).

### Plasmid constructions

The FLAG-tagged human METTL3 (Addgene, Cat. #53739), METTL14 (Addgene, Cat. #53740), ALKBH5 (Sino Biological, Cat. # HG24078-CF), and FTO (Sino Biological, Cat. # HG12125-NF) expression plasmids were purchased. The open reading frames of the BPIV3-M, -N, SeV-M, NiV-M, MeV-M, N, P, and hPIV3-M, -N, and -P genes were cloned into the eukaryotic expression plasmid pCAGGS or pcDNA. An HA tag was added to the N-terminus of SeV-M, NiV-M, MeV-M, and hPIV3-M. The BPIV3-M constructs with leucine substitution at residues L106 and L107 (L106A/L107A) in the NES and with lysine substitution at residues K258 (K258R) at the ubiquitination site of BPIV3-M, were generated by overlap PCR and were cloned into pCAGGS. The point mutants in NES and at the ubiquitination site of paramyxovirus M have been described previously [16]. The BPIV3-N constructs with the mutations for the disruption of m6A (non-m6A) sites at the nucleotide positions of 872 and 1146 (N872/1146), or 1372, 1427, and 1443 (N1372/1427/1443), and all positions (N-all) were also generated by overlap PCR and were cloned into pCAGGS. These M-L106A/L107A, M-K258R, and non-m6A mutations in the N gene were also inserted into the N gene in pUC57, which contained the complete genome of BPIV3 with the EGFP gene, as described previously [50]. The METTL3 genes lacking each domain were cloned into the pCAGGS vector. A BPIV3 minigenome plasmid encoding the NanoLuc luciferase reporter gene was constructed in the antigenomic orientation. The NanoLuc ORF was placed between the gene start signal derived from the BPIV3 N gene and the gene end signal from the L gene, and flanked by the BPIV3 leader and trailer sequences. The minigenome is set under the control of the T7 RNA polymerase promoter, and the transcript, expressed as a negative-sense RNA, is cleaved precisely at both ends by a hammerhead ribozyme and a hepatitis delta virus ribozyme. The construct was cloned into the pUC57 vector. The minigenome plasmid was used for BPIV3 minigenome assays in combination with the support plasmids expressing the BPIV3 N, P, and L proteins. For in vitro protein synthesis using a wheat germ cell-free expression system, a series of deletion mutants of the BPIV3 M gene were cloned into the pEU vector with N-terminal FLAG and GST tags.

### Reverse genetics for the rescue of recombinant BPIV3

BPIV3 wt and mutants were generated using a reverse genetics system with viral genomic RNA expression plasmids and viral protein support plasmids. Briefly, BHK/T7-9 cells were transfected with the BPIV3 genomic expression plasmid and the support plasmids pCAGGS-BPIV3-N, -P, and -L using Lipofectamine LTX (Thermo Fisher Scientific, Cat. #15338100). At 72 hpt, the supernatant and cells were harvested and co-cultured with fresh MDBK cells. When the EGFP fluorescent signal spread throughout the cells, the supernatant was collected and stored at -80°C until use.

### Virus titration

After infection with BPIV3 at a multiplicity of infection (MOI) of 1, cell culture supernatants were collected at various time points. To determine the viral titer, a 10-fold dilution series of the culture supernatant was prepared and MDBK cells seeded in 96-well plates were inoculated with the diluted viral solution. At 48–96 hpi, virus titers were calculated with TCID_50_ (50% tissue culture infectious dose) using EGFP fluorescence as an indicator.

### In vitro synthesis of recombinant M proteins using a wheat germ cell-free system

Recombinant M protein deletion mutants were synthesized using a wheat germ cell-free expression system (WEPRO7240G; CellFree Sciences) according to the manufacturer’s instructions. Plasmids encoding FLAG- and GST-tagged M protein deletion mutants cloned into the pEU vector were used as templates for in vitro transcription. A 20 µL transcription reaction mixture was prepared and incubated at 37°C for 6 h using SP6 RNA polymerase to generate capped mRNA. For translation, 20.8 µL of translation reaction mixture was prepared with WEPRO7240G and subjected to a bilayer translation reaction at 16°C for 20 h. After translation, the reaction mixture was gently mixed and collected. The synthesis of recombinant M proteins was confirmed by western blotting using an anti-FLAG Ab. The synthesized M proteins were subsequently used for a GST pull-down assay.

### Western blotting and coimmunoprecipitation

293T, A549, and HeLa cells seeded in 6-well plates were infected with rBPIV3-EGFP or mutants at an MOI of 1–5 or co-transfected with the METTL3 and m6A factors expression plasmids and BPIV3-N or BPIV3-M expression plasmids using TransIT-LT1 (Mirus, Cat. #MIR2300). When the virus was superinfected, BPIV3 was infected with MOI 1–5 at 24 hpt. After 48 h of transfection, cells were harvested, lysed in RIPA buffer, and subjected to western blotting. For co-immunoprecipitation, a mouse anti-FLAG Ab was added to the cell supernatant, lysed in RIPA buffer, and rotated at 4°C for 1 h. Protein A/G beads (Santa Cruz Biotechnology, Cat. #sc-2003) were then added to the supernatant, which was rotated at 4°C for 1 h. After washing, the sample buffer was added to the beads and subjected to western blotting. GST pull-down assays were performed as follows. The cells were transfected with a FLAG-METTL3 expression plasmid and harvested at 48–72 hpt. Cells were lysed in RIPA buffer, and the clarified supernatant was collected. The recombinant FLAG-GST-tagged M protein or its deletion mutants synthesized in a wheat germ cell-free system were incubated with glutathione-sepharose beads at 4°C, followed by washing with RIPA buffer. The FLAG-METTL3 lysate was then added to the beads and incubated again at 4°C. After washing with RIPA buffer, bound proteins were subjected to western blotting. METTL3 and recombinant M proteins were detected using an anti-FLAG Ab.

### Minigenome assay

293T cells were seeded in 6-well plates and transfected with 0.5 μg of BPIV3 minigenome plasmid, 0.4 μg of pCAGGS-BPIV3-N, 0.2 μg of pCAGGS-BPIV3-P, 0.4 μg of pCAGGS-BPIV3-L, and 1 μg of T7 polymerase expression plasmid using TransIT-LT1. At 48 h post-transfection, NanoLuc activity was measured using the Nano-Glo Luciferase Assay System (Promega, Cat. #N1120) on a GloMax Discovery System (Promega).

### Indirect immunofluorescence

A549 and HeLa cells seeded on coverslips were co-transfected with BPIV3-N and BPIV3-M expression plasmids, along with METTL3 and METTL14 expression plasmids, using Lipofectamine LTX. For virus superinfection, BPIV3 was inoculated at an MOI of 1 at 24 hpt. At 24–48 hpi, the cells were fixed in 10% buffered formalin solution and permeabilized with 0.5% Triton-X 100. After blocking, the cells were incubated with primary Abs, followed by incubation with secondary Abs conjugated with Alexa Fluor 488, 594, and 647 (Molecular Probes). The following primary antibodies were used: mouse anti-double-strand RNA mAb, rabbit anti-METTL3 pAb, mouse anti-m6A mAb, mouse anti-FLAG mAb, rabbit anti-FLAG Ab, mouse MeV-M mAb, rabbit anti-BPIV3-N or M pAb, and mouse anti-HA tag mAb. Nuclear staining was performed with 4′,6′-diamidino-2-phenylindole (DAPI). Cells were observed with a laser scanning confocal microscope (Olympus, FV3000). Colocalization efficiency was analyzed using the ImageJ Fiji software. The Manders’ coefficients were calculated using the Coloc 2 plugin to quantify the degree of colocalization between two fluorescent signals. Analyses were performed on 6–9 infected cells per condition. To evaluate the subcellular localization of METTL3 during BPIV3 infection or M coexpression, quantitative analysis was performed based on immunofluorescence microscopy. For each condition, analysis was conducted across more than 90 randomly selected fields, focusing on METTL3-positive cells that were also positive for M or N proteins. Cells were categorized as either “nucleus only” or “cytoplasmic or nucleus” based on the distribution of METTL3 signal. The number of cells in each category was counted in three independent experiments, and the relative proportions were calculated and presented as percentages.

### In situ PLA

An in-situ proximity ligation assay (PLA) was performed according to the manufacturer’s instructions (Duolink, Sigma-Aldrich). HeLa cells were infected with rBPIV3-EGFP at an MOI of 1 and fixed at 24–48 hpi, followed by permeabilization and blocking. Cells were incubated with mouse anti-m6A Ab and rabbit anti-N Ab for 1 h. After washing, species-specific PLA probes (anti-mouse PLUS and anti-rabbit MINUS) were applied. Phosphorylated connector oligonucleotides were hybridized to the PLA probes and ligated to form a circular DNA template when the probes were in close proximity (<40 nm). The template was subsequently amplified by rolling-circle amplification with DNA polymerase and detected with fluorescently labeled complementary oligonucleotides (Duolink Detection Kit Red). For dsRNA detection, mouse anti-dsRNA Ab was directly labeled using a FlexAble mouse IgG2a labeling kit (Proteintech). PLA signals were visualized by confocal microscopy, and the number of PLA foci was quantified using the Analyze Particle plug-in in ImageJ software. Colocalization of PLA signals with dsRNA or EGFP was quantified using Manders’ colocalization coefficient with the Coloc2 plugin in Fiji/ImageJ. Analyses were performed on 10 individual infected cells per condition from two independent experiments.

### Drug treatments and gene knock-down by siRNA

For STM2457 drug treatment, HeLa cells were pre-treated with 7.5 μM STM2457 24 h before virus infection. The cells were cultured in the presence or absence of the drug during virus infection. Supernatants were collected at 72 hpi and TCID_50_ was measured using MDBK. To examine the cytotoxicity of STM2457, cell viability after drug treatment was measured using CellTiter-Glo Luminescent Cell Viability Assay (Promega, Cat. #G7570). For treatment with LMB, HeLa cells were transfected with METTL3 expression plasmid. At 24 hpt, the cells were infected with BPIV3 at an MOI of 1, and 5 ng/mL of LMB was added at 4 hpi. At 48 hpi in the presence of LMB, the cells were fixed, and the subcellular localization of METTL3, and BPIV3-N was observed by confocal laser microscopy. For ActD treatment, 293T cells were transfected with BPIV3-N and non-m6A N mutant expression plasmids. At 48 hpt, the cells were treated with 5 μg/mL ActD for 9 h, and then, the cells were harvested and total RNA from the transfected cells was purified using NucleoSpin RNA (Takara Bio, Cat. #740955). For the knock-down of the Exportin-1 gene, HeLa cells were transfected with 50 nM siRNA for Exportin-1 (Santa Cruz Biotechnology, Cat. #sc-35116) and incubated for 48 h. The cells were harvested and subjected to western blotting and immunofluorescence.

### RNA immunoprecipitation (RIP) assay

RIP assay was performed using the RiboCluster Profiler RIP-Assay kit (MBL, Cat. # RN1001) according to the manufacturer’s instructions. Briefly, 293T cells were transfected with METTL3 expression plasmid. At 24 hpt, the cells were infected with BPIV3 at an MOI of 1. At 48 hpi, the cells were harvested and subjected to RIP assay; METTL3-viral RNA and complexes were immunoprecipitated from cell extracts using FLAG antibody or Myc antibody as control IgG, and high-quality viral RNA was purified. After viral RNA purification, cDNA from viral mRNA was synthesized by reverse transcription reaction with oligo dT primer using PrimeScript High Fidelity RT-PCR Kit (TaKaRa Bio, Cat. #R022A). The amount of N mRNA was quantified by qPCR using THUNDERBIRD Next SYBR qPCR Mix (TOYOBO, Cat. # QPX-201). For quantification, N gene-specific primer set (For: 5’-GCCTATGCCAACCCAGAATTA -3’, Rev: 5’-CTTAACTACGAGCCCACCATAC -3’) was used to calculate relative values to input RNA using the ΔΔCt method.

### Potential m6A prediction by sequence-based RNA adenosine methylation site predictor (SRAMP)

The m6A-modification sites in the *N* gene and other viral protein-coding genes of the BPIV3 BN-1 strain (Accession No. AB770484) were predicted via the SRAMP installed on a local computer [51]. The Full transcript mode was selected as a prediction mode. The score was evaluated by combining the binary score and the spectrum score.

### m6A RNA immunoprecipitation (MeRIP)

For the analysis of m6A modification of the BPIV3-N gene, 293T cells were infected with BPIV3 at an MOI of 1. At 72 hpi, infected cells were harvested, and total RNA was purified according to the manufacturer’s instructions using NucleoSpin RNA. The 20 μg of total RNA was used to purify mRNA using OligotexdT30 < Super> mRNA Purification Kit (From Total RNA) (TaKaRa BIO, Cat. #9086). MeRIP was performed with EpiQuik CUT&RUN m6A RNA Enrichment (MeRIP) Kit (EPIGENTEK, Cat. #9086P-9018–24) according to the manufacturer’s instructions using 5 μg of purified mRNA. The m6A-modified mRNA was immunoprecipitated with m6A-specific antibodies and magnetic beads. Followed by cleavage of the captured mRNA with the Cleavage enzyme, captured m6A mRNA was eluted from magnetic beads and purified. After mRNA purification, cDNA was synthesized by reverse transcription reaction using PrimeScript RT reagent Kit (Perfect Real Time) (TaKaRa BIO, Cat. #RR037A). The m6A sites in N mRNA were then identified by qPCR using THUNDERBIRD Next SYBR qPCR Mix. Primer sets were designed to target nucleotide (nt) 872, nt 1164, nt 1372, nt 1427, and nt 1443, sites with predicted high m6A scores by SRAMP. The nt 327, a low-scoring non-m6A site, was also chosen for negative control. Each primer set for amplification of 80–120 bp amplicons was designed as follows: m6A N872, For:5’- TGCAGGTCTTGCCTCATTT -3’, Rev: 5’- CTATCAGTGCCTTGAGCCTATT 3’, m6A N1146, For: 5’- TGATGCTGAATCGCAGATGAG -3’, Rev: 5’- GGGTTTGTGGAAGGTTGTATCT -3’, m6A N1372-1443, For: 5’- AGGAGTGATACCCAGACTGAA -3’, Rev: 5’- GGTCGCTCTCTGTTTCCTCTTT -3’, non-m6A N327, For: 5’- GCCTATGCCAACCCAGAATTA -3’, Rev: 5’- CTTAACTACGAGCCCACCATAC -3’. The relative value of each to the value of the input RNA level was calculated using the ΔΔCt method.

For analysis of IFN-β m6A modification, 293T cells were transfected with IFN-β + 3’UTR expression plasmid. At 48 hpt, the cells were harvested, and total RNA was purified using the same NucleoSpin RNA kit. MeRIP was performed using the same EpiQuik CUT&RUN m6A RNA Enrichment Kit following identical procedures as described above. Primer sets were designed to flank the two m6A modification sites in IFN-β mRNA CDS as described previously [33] using the following primer set: For: 5’- GAAGGCCAAGGAGTACAGTC -3’, Rev: 5’- GGAGGTAACCTGTAAGTCTGTTAAT-3’.

### N mRNA stability

293T cells were transfected with wt-N, N-872–1146, N1372-1443, and N-all mutant expression plasmids. At 48 hpt, the cells were treated with 5 μg/mL ActD for 9 h. After drug treatment, the total RNA was purified using NucleoSpin RNA and cDNA was synthesized by reverse transcription reaction using ReverTra Ace qPCR RT Master Mix with gDNA Remover (TOYOBO, Cat. #FSQ-201). The amount of N mRNA was quantified by qPCR using THUNDERBIRD Next SYBR qPCR Mix. For quantification, the N gene-specific primer set described above was used to calculate relative values to β-actin mRNA using the ΔΔCt method. Primer set for β-actin was designed as follows: For: -5’ ACCAACTGGGGACGACATGGGAGAAA -3’, Rev: 5’- TAGCACAGCCTGGATAGCAACGTA -3’.

### RNA-Seq and data analysis

A549 cells were transfected with the M expression plasmid and an empty vector. At 48 hpt, the cells were treated with 10 μg/ml poly (I:C) for 24 h, and total RNA was extracted from the cells using MagExtractor -RNA- (TOYOBO, Cat. #NPK-601) and subjected to RNA sequencing (RNA-seq) and cDNA synthesis followed by qPCR of IFN-β mRNA. Total RNA was measured by Quantus Fluorometer and QuantiFluor RNA system (Promega, Cat. #E3310). RNA quality was then verified using a 5300 Fragment Analyzer System and an Agilent HS RNA Kit (Agilent Technologies, Cat. #DNF-472–0500). Subsequently, RNA libraries were constructed using the MGIEasy RNA Directional Library Prep Set (MGI Tech, Cat. #1000006386). Then, the concentration of the prepared library solutions was quantified using a Qubit 3.0 Fluorometer and the dsDNA High Sensitivity Assay Kit (Thermo Fisher Scientific, Cat. #Q32851). Furthermore, library quality was assessed with the Agilent 2100 Bioanalyzer and the High Sensitivity DNA Kit (Agilent Technologies, Cat. #5067–4626). Circularized DNA was generated from the prepared library using the MGIEasy Circularization Kit (MGI Tech, Cat. #1000005259). Afterward, DNA Nanoballs were produced using the DNB Rapid Make Reagent Kit (MGI Tech, Cat. #1000028453). Sequencing analysis was performed using DNBSEQ-G400 at 2x150 bp with the addition of Primer supplied with the High-Throughput Pair-End Sequencing Primer Kit (App-D, Cat. #1000020832). Normalization and identification of differentially expressed genes (DEGs) were performed using the R packages TCC (version 1.18.0) and DESeq (ver. 1.30.0), respectively. DEG visualization with volcano plots was performed on Prism 10. (version 10.1.1). The canonical pathway analysis and upstream analysis of DEGs were performed on the gene list from the QIAGEN Knowledgebase using Ingenuity Pathway Analysis (IPA) (Content version: 127006219, Release Date: 2024-11-17; Qiagen, Hilden, Germany).

### Quantification of IFN-β mRNA by qPCR

A549 cells transfected with M expression plasmid or infected with BPIV3, and METTL3-KD HeLa cells infected with BPIV3, were harvested at 48 hours post-transfection or post-infection. Total RNA was extracted using the MagExtractor -RNA- kit (TOYOBO) according to the manufacturer’s instructions. The cDNA was synthesized from the extracted RNA using ReverTra Ace qPCR RT Master Mix with gDNA Remover. Quantitative PCR (qPCR) was then performed using THUNDERBIRD Next SYBR qPCR Mix (TOYOBO) to determine the relative levels of IFN-β mRNA. Primer sets for IFN-β quantification were as follows: For: -5’ CTTGGATTCCTACAAAGAAGCAGC-3’ and Rev: 5’- TCCTCCTTCTGGAACTGCTGCA-3’. Relative expression levels of IFN-β mRNA were calculated using the ΔΔCt method, normalized to β-actin mRNA.

### Quantification of BPIV3 genome and antigenome RNA

293T cells were transfected with either an empty vector or an N protein expression plasmid. At 24 hours post-transfection, cells were infected with wt BPIV3 or the N1372/1427/1443 mutant virus at an MOI of 1. Total RNA was extracted at 48 hpi using the MagExtractor -RNA- kit (TOYOBO) according to the manufacturer’s protocol. Strand-specific primers used for cDNA synthesis were as follows: BPIV3 genome cDNA primer: 5’-AAGAGAAGAGACTTGTTTGGGAATA-3’ and BPIV3 antigenome cDNA primer: 5’-TATTCCCAAACAAGTCTCTTCTCTT-3’. RT-PCR was performed with the PrimeScript High Fidelity RT-PCR Kit (TaKaRa Bio) using these strand-specific primers to synthesize cDNA. qPCR targeting the N gene was then carried out using THUNDERBIRD Next SYBR qPCR Mix (TOYOBO) with the following primers: For: 5’-GCCTATGCCAACCCAGAATTA-3’ and Rev: 5’-CTTAACTACGAGCCCACCATAC-3’. Relative expression levels of genome and antigenome RNA were calculated using the ΔΔCt method with normalization to β-actin mRNA.

### Statistical analysis

All statistical analyses were performed using GraphPad Prism 9 software (version 9.4.1). Data are presented as the mean ± standard deviation of the mean, as indicated. For comparisons between two groups, an unpaired two-tailed Student’s t-test was used. For multiple group comparisons, one-way or two-way analysis of variance (ANOVA) followed by Dunnett’s or Šídák’s multiple comparisons test was applied, depending on the experimental design. Each experiment was independently performed at least three times (biological replicates), and technical replicates were included as appropriate. A *p*-value < 0.05 was considered statistically significant. In DESeq, a difference with a *q*-value (false discovery rate, FDR) < 0.05 was considered statistically significant. In IPA, *p*-values were calculated using Fisher’s exact test, and values <0.05 were considered statistically significant.

## Supporting information

S1 Fig **(A)** HeLa cells were infected with rBPIV3-EGFP at an MOI of 1 and fixed at 24–48 hpi, followed by permeabilization and blocking. Primary antibodies against m6A (mouse) and N protein (rabbit) were incubated, and in situ PLA was performed using Duolink In Situ PLA probes (anti-mouse PLUS and anti-rabbit MINUS), followed by ligation and rolling circle amplification. After PLA, cells were stained with an anti-dsRNA antibody directly labeled with a fluorescent dye (405 nm; FlexAble labeling kit). Representative images from two independent experiments are shown. White boxes indicate regions magnified in the upper-right insets. **(B)** Quantification of PLA signals was performed in 10 cells per condition using the Analyze Particles plugin in Fiji/ImageJ software. Bars represent the mean ± SD from two independent experiments. **(C)** Colocalization of PLA signals with dsRNA or EGFP was quantified using Manders’ colocalization coefficient (Coloc2 plugin, Fiji/ImageJ). Data represent analyses of 10 individual infected cells per condition from two independent experiments. Asterisks indicate statistical significance (*p < 0.05).
(DOCX)

S2 Fig 293T cells were transfected with plasmids expressing METTL3-FLAG, METTL14-FLAG, FTO-FLAG, ALKBH5-FLAG, or an empty vector control. At 24 h post-transfection, the cells were infected with rBPIV3-EGFP at an MOI of 1. At 72 hpi, cells were harvested and subjected to western blotting. Viral N protein was detected using an anti-BPIV3-N antibody, while m6A regulatory factors were detected using an anti-FLAG antibody (A). HeLa cells stably expressing control shRNA (Control), shRNA targeting METTL3 (METTL3 KD), or shRNA targeting METTL14 (METTL14 KD) were infected with rBPIV3-EGFP at an MOI of 1. At 48 h post-infection, cells were harvested and subjected to western blotting to detect BPIV3-N, endogenous METTL3, or endogenous METTL14 using specific antibodies.
(TIF)

S3 Fig A549 cells were transfected with a FLAG-METTL3 expression plasmid and, at 24 h post-transfection, infected with rBPIV3-EGFP at an MOI of 1. At 48 h post-infection, cells were fixed and co-stained with anti-BPIV3-N antibody and anti-FLAG antibody to detect METTL3. Images were acquired at low magnification using a confocal laser-scanning microscope. Arrows indicate METTL3-positive infected cells, and arrowheads indicate METTL3-positive uninfected cells. Nuclei were counterstained with DAPI.
(DOCX)

S4 Fig 293T cells were transfected with METTL3 expression plasmid, and at 24 h post-transfection, infected with BPIV3 at an MOI of 1. At 48 h post-infection, cells were subjected to UV crosslinking to covalently stabilize interactions between RNA and RNA-binding proteins. Cells were then harvested and lysed. Lysates were immunoprecipitated with anti-dsRNA antibody or control IgG. The immunoprecipitates were analyzed by western blotting using anti-BPIV3-N antibody and anti-FLAG antibody for METTL3 detection.
(DOCX)

S5 Fig Absence of METTL3 nuclear export during co-expression with viral nucleocapsid protein. HeLa cells were singly transfected with METTL3 expression plasmid or cotransfected with METTL3 and N expression plasmids. At 48 h post-transfection, the cells were fixed and stained with anti-FLAG antibody (Ab) only for METTL3 or costained with anti-BPIV3-N Ab and anti-FLAG Ab.
(DOCX)

S6 Fig HeLa cells were cotransfected with METTL3 and BPIV3-M expression plasmids. At 24, 48, and 72 hours post-transfection (hpt), cells were fixed and immunostained with an anti-FLAG antibody to detect METTL3 and an anti-M antibody to detect BPIV3-M (A). For classification-based quantification, more than 30 METTL3/M double-positive cells per condition were randomly selected and classified into two localization patterns (“nucleus only” or “cytoplasmic or nucleus”). The number of cells in each pattern was counted, and the results are presented as the percentage of total cells analyzed (B). All experiments were performed independently three times. Asterisks indicate statistically significant differences (*p < 0.05).
(DOCX)

S7 Fig Common mechanism of M-mediated METTL3 nuclear export among paramyxoviruses. HeLa cells were cotransfected with Sendai virus M (SeV-M), human parainfluenza virus type 3 M (hPIV3-M) +N + P, Nipah virus M (NiV-M), and Measles virus M (MeV-M) +N + P along with METTL3. At 48 h post-transfection, the cells were costained with anti-HA antibody (Ab) for SeV-M, HPIV3-M, and NiV-M, anti-MeV-M Ab and anti-FLAG Ab for METTL3. Nuclei were stained with DAPI (A). For quantification, more than 40 METTL3/M double-positive cells were randomly selected and categorized into two subcellular localization patterns: nucleus only (B) or cytoplasmic or nucleus (C). The number of cells in each category was counted, and the results are presented as the percentage of total cells analyzed. Asterisks indicate statistically significant differences (*p < 0.05). All experiments were performed independently three times.
(DOCX)

S8 Fig Prediction of m6A modification sites in BPIV3 protein genes. The m6A probability scores across the open reading frames (ORFs) of all BPIV3 proteins (N, P, M, F, HN, and L) were calculated using the Sequence-based RNA Adenosine Methylation site Predictor (SRAMP). The blue graph represents the m6A probability score at each position, with the horizontal red line indicating the threshold score of 0.5. The analysis revealed several regions with high m6A modification potential, particularly within the N and P gene ORF regions.
(DOCX)

S9 Fig Effects of N-1372/1427/1443 mutations on viral replication and RNA synthesis. BHK/T7-9 cells were cotransfected with a BPIV3 minigenome plasmid harboring a T7 promoter and NanoLuc gene together with support plasmids encoding N (wild-type [wt-N] or mutant [N-1372/1427/1443 or N-all]), P, and L proteins. At 48 h post-transfection (hpt), cells were lysed, and NanoLuc luciferase activity was measured. Luciferase activity obtained with mutant N proteins was normalized to that obtained with wt-N. Data are expressed as relative light units (A). A549 and HeLa cells were infected with either wt or N-1372/1427/1443 mutant BPIV3 at an MOI of 5. At 24 h post-infection (hpi), cells were harvested, and N and M proteins were detected by western blotting (B). 293T cells were transfected with either an N expression plasmid or an empty vector. At 24 hpt, cells were infected with either wt BPIV3 or the N-1372/1427/1443 mutant virus at an MOI of 0.1–1. At 24–48 hpi, culture supernatants and cells were collected. Viral titers in the supernatants were determined by the TCID_50_ assay (C). Total RNA was extracted from infected cells and strand-specific RT-PCR was performed to generate cDNA corresponding to genomic or antigenomic RNA. qPCR using N-specific primers was then carried out to quantify viral genome (D) and antigenome (E) levels. All experiments were performed independently three times. Asterisks indicate statistically significant differences (* *p* < 0.05); ns, not significant.
(DOCX)

S10 Fig Impact of METTL3 expression and M-K258R mutation on IFN-β mRNA expression and m6A modification. HeLa cells were transfected with a FLAG-METTL3 expression plasmid and, at 24 hours post-transfection (hpt), infected with either wt-BPIV3 or the M-K258R mutant at an MOI of 1. At 48 hours post-infection (hpi), cells were fixed and co-stained with anti-FLAG antibody for METTL3 and anti-M antibody (A). For classification-based quantification, more than 40 METTL3/M double-positive cells per condition were randomly selected and categorized into two subcellular localization patterns (“nucleus only” or “cytoplasmic or nucleus”). The number of cells in each category was counted, and the results are presented as the percentage of total cells analyzed (B). A549 cells were infected with wt-BPIV3 or the M-K258R mutant at an MOI of 1, and total RNA was extracted from infected cells at 24–48 hpi. After reverse transcription, IFN-β mRNA levels were quantified by qPCR (C). Total RNA extracted from infected or mock-infected cells was subjected to m6A RNA immunoprecipitation using an m6A-specific antibody. IFN-β m6A modification efficiency was determined by qPCR using primer sets targeting m6A sites within IFN-β mRNA, with enriched RNA as the template (D). A549 cells were transfected with a METTL3 expression plasmid or an empty vector, and were infected with BPIV3 at an MOI of 1 at 24 hpt. At 48 hpi, cells were harvested, total RNA was extracted, reverse-transcribed, and IFN-β mRNA levels were quantified by qPCR (E). METTL3-knockdown (KD) or control shRNA HeLa cells were infected with BPIV3 at an MOI of 1. At 48 hpi, cells were processed as in panel E to quantify IFN-β mRNA levels (F). All experiments were performed independently three times. Asterisks represent statistically significant differences (* *p* < 0.05); ns, not significant.
(DOCX)

S1 Data All quantitative data underlying the Figs and original western blot images are provided in this file. (XLSX)

## References

[1] Tsai K, Cullen BR. Epigenetic and epitranscriptomic regulation of viral replication. Nat Rev Microbiol. 2020;18(10):559–70. doi: 10.1038/s41579-020-0382-3 32533130 PMC7291935

[2] Mersinoglu B, Cristinelli S, Ciuffi A. The Impact of Epitranscriptomics on Antiviral Innate Immunity. Viruses. 2022;14(8):1666. doi: 10.3390/v14081666 36016289 PMC9412694

[3] Ribeiro DR, Nunes A, Ribeiro D, Soares AR. The hidden RNA code: implications of the RNA epitranscriptome in the context of viral infections. Front Genet. 2023;14:1245683. doi: 10.3389/fgene.2023.1245683 37614818 PMC10443596

[4] He PC, He C. m6 A RNA methylation: from mechanisms to therapeutic potential. EMBO J. 2021;40(3):e105977. doi: 10.15252/embj.2020105977 33470439 PMC7849164

[5] Malovic E, Ealy A, Kanthasamy A, Kanthasamy AG. Emerging Roles of N6-Methyladenosine (m6A) Epitranscriptomics in Toxicology. Toxicol Sci. 2021;181(1):13–22. doi: 10.1093/toxsci/kfab021 33616673 PMC8599717

[6] Desrosiers R, Friderici K, Rottman F. Identification of methylated nucleosides in messenger RNA from Novikoff hepatoma cells (RNA methylation/RNA processing/methylnucleoside composition). 1974. 10.1073/pnas.71.10.3971 PMC434308 4372599

[7] McFadden MJ, Horner SM. N6-Methyladenosine Regulates Host Responses to Viral Infection. Trends Biochem Sci. 2021;46(5):366–77. doi: 10.1016/j.tibs.2020.11.008 33309325 PMC8052259

[8] Dominissini D, Moshitch-Moshkovitz S, Schwartz S, Salmon-Divon M, Ungar L, Osenberg S, et al. Topology of the human and mouse m6A RNA methylomes revealed by m6A-seq. Nature. 2012;485(7397):201–6. doi: 10.1038/nature11112 22575960

[9] Meyer KD, Saletore Y, Zumbo P, Elemento O, Mason CE, Jaffrey SR. Comprehensive analysis of mRNA methylation reveals enrichment in 3’ UTRs and near stop codons. Cell. 2012;149(7):1635–46. doi: 10.1016/j.cell.2012.05.003 22608085 PMC3383396

[10] Batista PJ. The RNA Modification N6-methyladenosine and Its Implications in Human Disease. Genomics Proteomics Bioinformatics. 2017;15(3):154–63. doi: 10.1016/j.gpb.2017.03.002 28533023 PMC5487527

[11] Maity A, Das B. N6-methyladenosine modification in mRNA: machinery, function and implications for health and diseases. FEBS J. 2016;283(9):1607–30. doi: 10.1111/febs.13614 26645578

[12] Roundtree IA, Evans ME, Pan T, He C. Dynamic RNA Modifications in Gene Expression Regulation. Cell. 2017;169(7):1187–200. doi: 10.1016/j.cell.2017.05.045 28622506 PMC5657247

[13] Shi H, Wei J, He C. Where, When, and How: Context-Dependent Functions of RNA Methylation Writers, Readers, and Erasers. Molecular Cell. Cell Press; 2019. pp. 640–650. doi: 10.1016/j.molcel.2019.04.025 PMC6527355 31100245

[14] El Najjar F, Schmitt AP, Dutch RE. Paramyxovirus glycoprotein incorporation, assembly and budding: a three way dance for infectious particle production. Viruses. 2014;6(8):3019–54. doi: 10.3390/v6083019 25105277 PMC4147685

[15] Katoh H, Kimura R, Sekizuka T, Matsuoka K, Hosogi M, Kitai Y. Structural and molecular properties of mumps virus inclusion bodies. Sci Adv. 2024. 10.1126/sciadv.adr0359 PMC11623304 39642233

[16] Pentecost M, Vashisht AA, Lester T, Voros T, Beaty SM, Park A, et al. Evidence for ubiquitin-regulated nuclear and subnuclear trafficking among Paramyxovirinae matrix proteins. PLoS Pathog. 2015;11(3):e1004739. doi: 10.1371/journal.ppat.1004739 25782006 PMC4363627

[17] Hung C-T, Haas GD, Watkinson RE, Chiu H-P, Kowdle S, Stevens CS, et al. Paramyxovirus matrix proteins modulate host cell translation via exon-junction complex interactions in the cytoplasm. bioRxiv. 2024;:2024.09.05.611502. doi: 10.1101/2024.09.05.611502 39282406 PMC11398453

[18] Fearns R, Plemper RK. Polymerases of paramyxoviruses and pneumoviruses. Virus Res. 2017;234:87–102. doi: 10.1016/j.virusres.2017.01.008 28104450 PMC5476513

[19] Katoh H, Sekizuka T, Nakatsu Y, Nakagawa R, Nao N, Sakata M, et al. The R2TP complex regulates paramyxovirus RNA synthesis. PLoS Pathog. 2019;15(5):e1007749. doi: 10.1371/journal.ppat.1007749 31121004 PMC6532945

[20] Dolnik O, Gerresheim GK, Biedenkopf N. New Perspectives on the Biogenesis of Viral Inclusion Bodies in Negative-Sense RNA Virus Infections. Cells. 2021;10(6):1460. doi: 10.3390/cells10061460 34200781 PMC8230417

[21] Ma D, George CX, Nomburg JL, Pfaller CK, Cattaneo R, Samuel CE. Upon Infection, Cellular WD Repeat-Containing Protein 5 (WDR5) Localizes to Cytoplasmic Inclusion Bodies and Enhances Measles Virus Replication. J Virol. 2018;92(5):e01726-17. doi: 10.1128/JVI.01726-17 29237839 PMC5809725

[22] Yuan W, Hou Y, Wang Q, Lv T, Ren J, Fan L, et al. Newcastle disease virus activates methylation-related enzymes to reprogram m6A methylation in infected cells. Vet Microbiol. 2023;281:109747. doi: 10.1016/j.vetmic.2023.109747 37080085

[23] Xue M, Zhao BS, Zhang Z, Lu M, Harder O, Chen P, et al. Viral N6-methyladenosine upregulates replication and pathogenesis of human respiratory syncytial virus. Nat Commun. 2019;10(1):4595. doi: 10.1038/s41467-019-12504-y 31597913 PMC6785563

[24] Lu M, Zhang Z, Xue M, Zhao BS, Harder O, Li A, et al. N6-methyladenosine modification enables viral RNA to escape recognition by RNA sensor RIG-I. Nat Microbiol. 2020;5(4):584–98. doi: 10.1038/s41564-019-0653-9 32015498 PMC7137398

[25] Gokhale NS, McIntyre ABR, McFadden MJ, Roder AE, Kennedy EM, Gandara JA, et al. N6-Methyladenosine in Flaviviridae Viral RNA Genomes Regulates Infection. Cell Host Microbe. 2016;20(5):654–65. doi: 10.1016/j.chom.2016.09.015 27773535 PMC5123813

[26] Zhang X, Hao H, Ma L, Zhang Y, Hu X, Chen Z, et al. Methyltransferase-like 3 Modulates Severe Acute Respiratory Syndrome Coronavirus-2 RNA N6-Methyladenosine Modification and Replication. mBio. 2021;12(4):e0106721. doi: 10.1128/mBio.01067-21 34225491 PMC8437041

[27] Li Y, He X, Lu X, Gong Z, Li Q, Zhang L, et al. METTL3 acetylation impedes cancer metastasis via fine-tuning its nuclear and cytosolic functions. Nat Commun. 2022;13(1):6350. doi: 10.1038/s41467-022-34209-5 36289222 PMC9605963

[28] Wang YE, Park A, Lake M, Pentecost M, Torres B, Yun TE, et al. Ubiquitin-regulated nuclear-cytoplasmic trafficking of the Nipah virus matrix protein is important for viral budding. PLoS Pathog. 2010;6(11):e1001186. doi: 10.1371/journal.ppat.1001186 21085610 PMC2978725

[29] Nachury M V, Weis K. The direction of transport through the nuclear pore can be inverted. Cell Biology. 1999. Available: www.pnas.org 10.1073/pnas.96.17.9622 PMC22259 10449743

[30] Stade K, Ford CS, Guthrie C, Weis K. Exportin 1 (Crm1p) is an essential nuclear export factor. Cell. 1997;90(6):1041–50. doi: 10.1016/s0092-8674(00)80370-0 9323132

[31] Śledź P, Jinek M. Structural insights into the molecular mechanism of the m(6)A writer complex. Elife. 2016;5:e18434. doi: 10.7554/eLife.18434 27627798 PMC5023411

[32] Corbeski I, Vargas-Rosales PA, Bedi RK, Deng J, Coelho D, Braud E, et al. The catalytic mechanism of the RNA methyltransferase METTL3. Elife. 2024;12:RP92537. doi: 10.7554/eLife.92537 38470714 PMC10932547

[33] Winkler R, Gillis E, Lasman L, Safra M, Geula S, Soyris C, et al. m6A modification controls the innate immune response to infection by targeting type I interferons. Nat Immunol. 2019;20(2):173–82. doi: 10.1038/s41590-018-0275-z 30559377

[34] Liu J, Xu Y-P, Li K, Ye Q, Zhou H-Y, Sun H, et al. The m6A methylome of SARS-CoV-2 in host cells. Cell Res. 2021;31(4):404–14. doi: 10.1038/s41422-020-00465-7 33510385 PMC8115241

[35] Burgess HM, Depledge DP, Thompson L, Srinivas KP, Grande RC, Vink EI, et al. Targeting the m6A RNA modification pathway blocks SARS-CoV-2 and HCoV-OC43 replication. Genes Dev. 2021;35(13–14):1005–19. doi: 10.1101/gad.348320.121 34168039 PMC8247602

[36] Qiu W, Zhang Q, Zhang R, Lu Y, Wang X, Tian H, et al. N6-methyladenosine RNA modification suppresses antiviral innate sensing pathways via reshaping double-stranded RNA. Nat Commun. 2021;12(1):1582. doi: 10.1038/s41467-021-21904-y 33707441 PMC7952553

[37] Liu Y, You Y, Lu Z, Yang J, Li P, Liu L, et al. N 6-methyladenosine RNA modification-mediated cellular metabolism rewiring inhibits viral replication. Available: https://www.science.org 10.1126/science.aax4468 31439758

[38] Kennedy EM, Bogerd HP, Kornepati AVR, Kang D, Ghoshal D, Marshall JB, et al. Posttranscriptional m(6)A Editing of HIV-1 mRNAs Enhances Viral Gene Expression. Cell Host Microbe. 2016;19(5):675–85. doi: 10.1016/j.chom.2016.04.002 27117054 PMC4867121

[39] Kennedy EM, Courtney DG, Tsai K, Cullen BR. Viral Epitranscriptomics. J Virol. 2017;91(9):e02263-16. doi: 10.1128/JVI.02263-16 28250115 PMC5391447

[40] Courtney DG, Kennedy EM, Dumm RE, Bogerd HP, Tsai K, Heaton NS, et al. Epitranscriptomic Enhancement of Influenza A Virus Gene Expression and Replication. Cell Host Microbe. 2017;22(3):377-386.e5. doi: 10.1016/j.chom.2017.08.004 28910636 PMC5615858

[41] Norris MJ, Husby ML, Kiosses WB, Yin J, Saxena R, Rennick LJ. Measles and Nipah virus assembly: Specific lipid binding drives matrix polymerization. Sci Adv. 2022. 10.1126/sciadv.abn1440 PMC9299542 35857835

[42] Ueda H, Yamakawa N, Takeuchi K. Amino- and carboxyl-terminal ends of the bovine parainfluenza virus type 3 matrix protein are important for virion and virus-like particle release. Virology. 2021;561:17–27. doi: 10.1016/j.virol.2021.05.014 34130198

[43] Rawlinson SM, Zhao T, Rozario AM, Rootes CL, McMillan PJ, Purcell AW, et al. Viral regulation of host cell biology by hijacking of the nucleolar DNA-damage response. Nat Commun. 2018;9(1):3057. doi: 10.1038/s41467-018-05354-7 30076298 PMC6076271

[44] Wakata A, Katoh H, Kato F, Takeda M. Nucleolar Protein Treacle Is Important for the Efficient Growth of Mumps Virus. J Virol. 2022;96(19):e0072222. doi: 10.1128/jvi.00722-22 36135364 PMC9555161

[45] Aly A, Scott G, Calderon M, Haghighi AP. N6-Adenosine Methylation of SARS-CoV-2 5’-UTR Regulates Translation. bioRxiv. 2022;:2022.10.17.512569. doi: 10.1101/2022.10.17.512569 36299421 PMC9603819

[46] Imam H, Khan M, Gokhale NS, McIntyre ABR, Kim G-W, Jang JY, et al. N6-methyladenosine modification of hepatitis B virus RNA differentially regulates the viral life cycle. Proc Natl Acad Sci U S A. 2018;115(35):8829–34. doi: 10.1073/pnas.1808319115 30104368 PMC6126736

[47] Lichinchi G, Zhao BS, Wu Y, Lu Z, Qin Y, He C, et al. Dynamics of Human and Viral RNA Methylation during Zika Virus Infection. Cell Host Microbe. 2016;20(5):666–73. doi: 10.1016/j.chom.2016.10.002 27773536 PMC5155635

[48] Gokhale NS, McIntyre ABR, Mattocks MD, Holley CL, Lazear HM, Mason CE, et al. Altered m6A Modification of Specific Cellular Transcripts Affects Flaviviridae Infection. Mol Cell. 2020;77(3):542-555.e8. doi: 10.1016/j.molcel.2019.11.007 31810760 PMC7007864

[49] Selberg S, Blokhina D, Aatonen M, Koivisto P, Siltanen A, Mervaala E, et al. Discovery of Small Molecules that Activate RNA Methylation through Cooperative Binding to the METTL3-14-WTAP Complex Active Site. Cell Rep. 2019;26(13):3762-3771.e5. doi: 10.1016/j.celrep.2019.02.100 30917327

[50] Okura T, Tahara M, Otsuki N, Sato M, Takeuchi K, Takeda M. Generation of a photocontrollable recombinant bovine parainfluenza virus type 3. Microbiol Immunol. 2023;67(4):204–9. doi: 10.1111/1348-0421.13052 36609846

[51] Zhou Y, Zeng P, Li Y-H, Zhang Z, Cui Q. SRAMP: prediction of mammalian N6-methyladenosine (m6A) sites based on sequence-derived features. Nucleic Acids Res. 2016;44(10):e91. doi: 10.1093/nar/gkw104 26896799 PMC4889921

