# Supplementary material for: Paramyxovirus matrix protein redirects METTL3 for dual regulation of viral replication and immune evasion
Source: PLoS Pathog. 2025 Dec 1;21(12):e1013755. doi: 10.1371/journal.ppat.1013755 (PMC12680350; doi:10.1371/journal.ppat.1013755)
Supplement: S1 Fig — (A) HeLa cells were infected with rBPIV3-EGFP at an MOI of 1 and fixed at 24–48 hpi, followed by permeabilization and blocking. Primary antibodies against m6A (mouse) and N protein (rabbit) were incubated, and in situ PLA was performed using Duolink In Situ PLA probes (anti-mouse PLUS and anti-rabbit MINUS), followed by ligation and rolling circle amplification. After PLA, cells were stained with an anti-dsRNA antibody directly labeled with a fluorescent dye (405 nm; FlexAble labeling kit). Representative images from two independent experiments are shown. White boxes indicate regions magnified in the upper-right insets. (B) Quantification of PLA signals was performed in 10 cells per condition using the Analyze Particles plugin in Fiji/ImageJ software. Bars represent the mean ± SD from two independent experiments. (C) Colocalization of PLA signals with dsRNA or EGFP was quantified using Manders’ colocalization coefficient (Coloc2 plugin, Fiji/ImageJ). Data represent analyses of 10 individual infected cells per condition from two independent experiments. Asterisks indicate statistical significance (*p < 0.05). (DOCX) [file ppat.1013755.s001.docx]

**
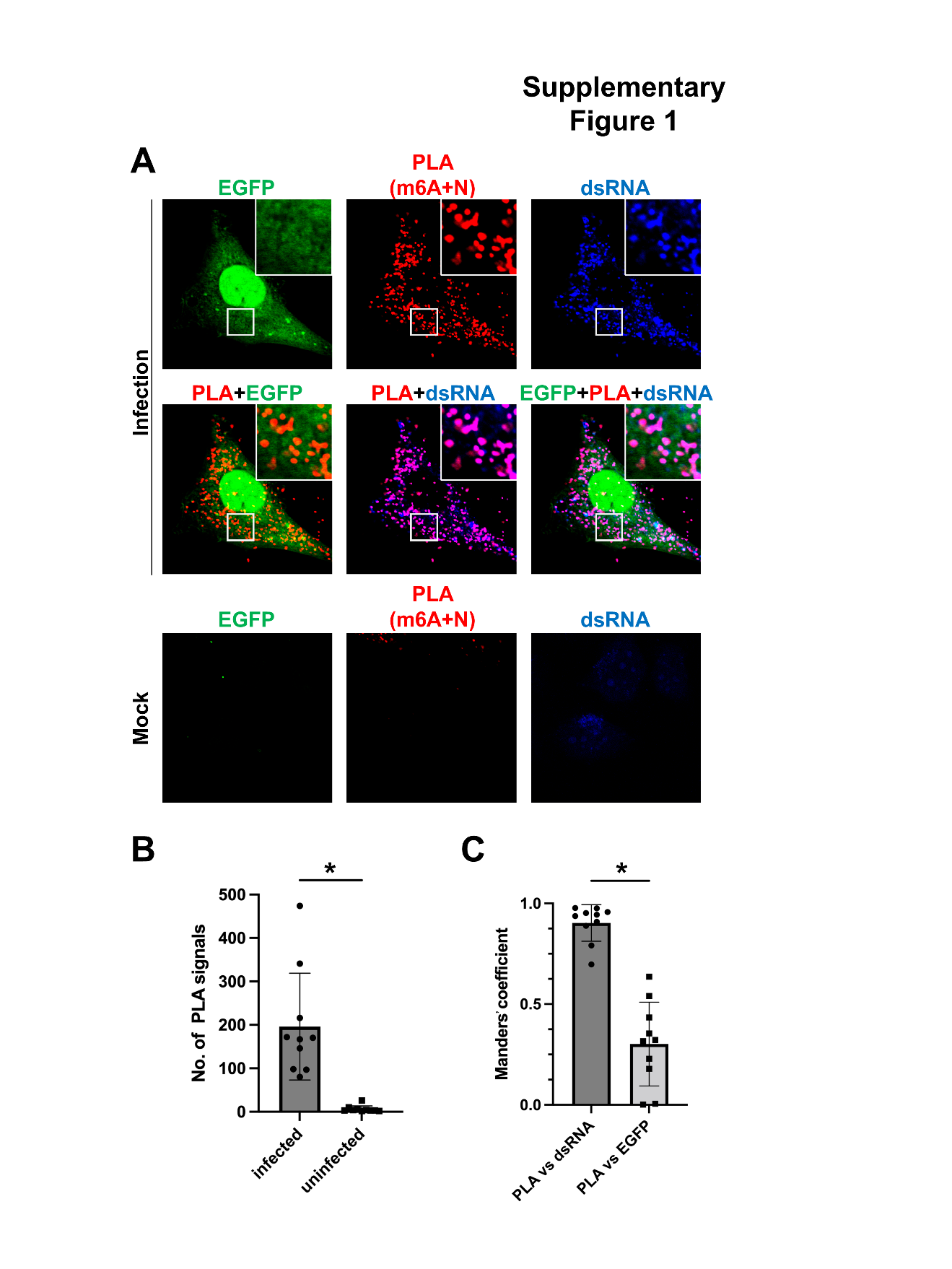
**

Supplementary Figure 1. (A) HeLa cells were infected with rBPIV3-EGFP at an MOI of 1 and fixed at 24–48 hpi, followed by permeabilization and blocking. Primary antibodies against m6A (mouse) and N protein (rabbit) were incubated, and in situ PLA was performed using Duolink In Situ PLA probes (anti-mouse PLUS and anti-rabbit MINUS), followed by ligation and rolling circle amplification. After PLA, cells were stained with an anti-dsRNA antibody directly labeled with a fluorescent dye (405 nm; FlexAble labeling kit). Representative images from two independent experiments are shown. White boxes indicate regions magnified in the upper-right insets. (B) Quantification of PLA signals was performed in 10 cells per condition using the Analyze Particles plugin in Fiji/ImageJ software. Bars represent the mean ± SD from two independent experiments. (C) Colocalization of PLA signals with dsRNA or EGFP was quantified using Manders’ colocalization coefficient (Coloc2 plugin, Fiji/ImageJ). Data represent analyses of 10 individual infected cells per condition from two independent experiments. Asterisks indicate statistical significance (**p* < 0.05)
