# Supplementary figures and images for: Paramyxovirus matrix protein redirects METTL3 for dual regulation of viral replication and immune evasion
Source: PLoS Pathog. 2025 Dec 1;21(12):e1013755. doi: 10.1371/journal.ppat.1013755 (PMC12680350; doi:10.1371/journal.ppat.1013755)

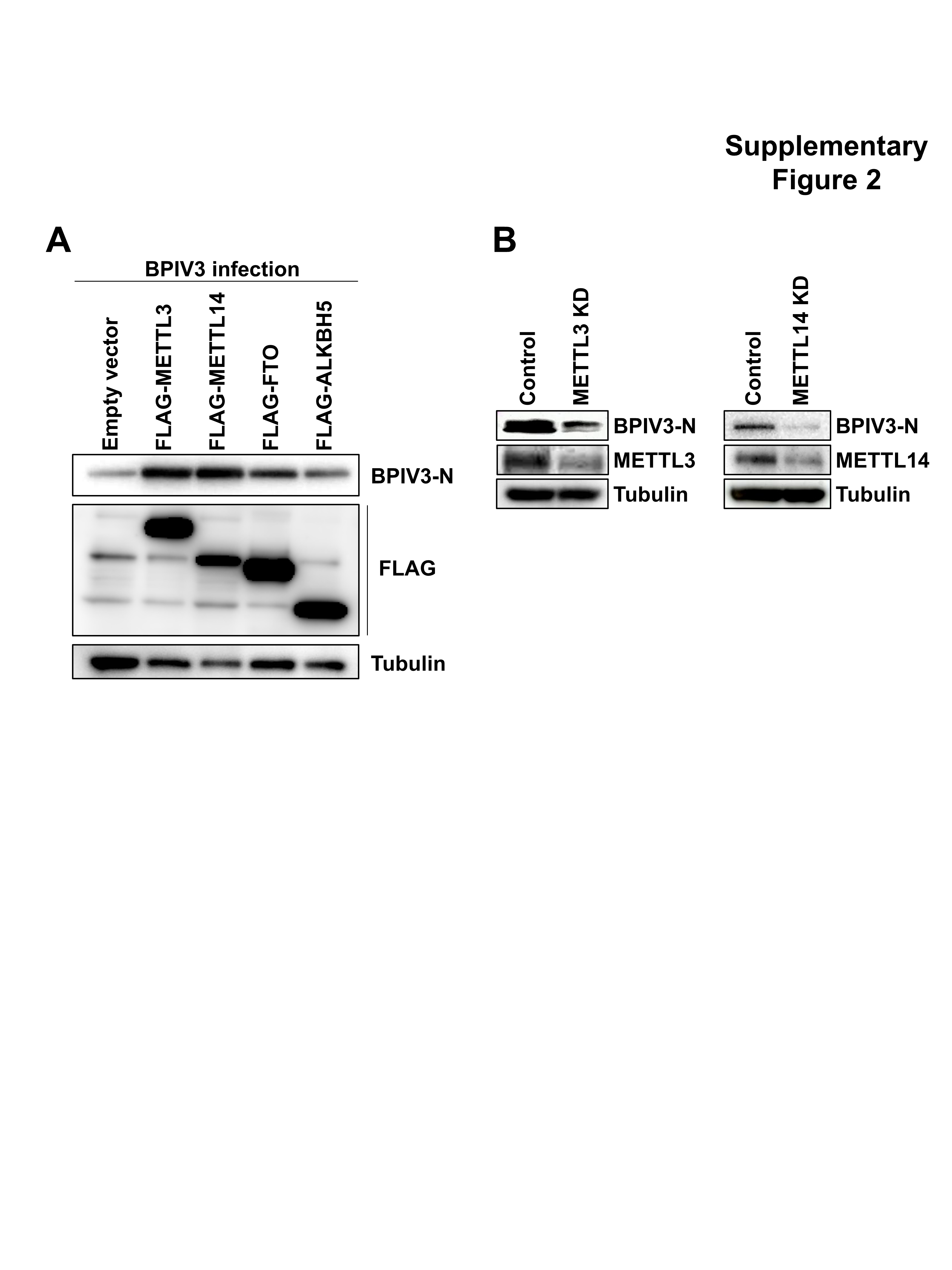

Supplement: S2 Fig — At 24 h post-transfection, the cells were infected with rBPIV3-EGFP at an MOI of 1. At 72 hpi, cells were harvested and subjected to western blotting. Viral N protein was detected using an anti-BPIV3-N antibody, while m6A regulatory factors were detected using an anti-FLAG antibody (A). HeLa cells stably expressing control shRNA (Control), shRNA targeting METTL3 (METTL3 KD), or shRNA targeting METTL14 (METTL14 KD) were infected with rBPIV3-EGFP at an MOI of 1. At 48 h post-infection, cells were harvested and subjected to western blotting to detect BPIV3-N, endogenous METTL3, or endogenous METTL14 using specific antibodies. (TIF) [file ppat.1013755.s002.tif]
