# Supplementary material for: Paramyxovirus matrix protein redirects METTL3 for dual regulation of viral replication and immune evasion
Source: PLoS Pathog. 2025 Dec 1;21(12):e1013755. doi: 10.1371/journal.ppat.1013755 (PMC12680350; doi:10.1371/journal.ppat.1013755)
Supplement: S3 Fig — At 48 h post-infection, cells were fixed and co-stained with anti-BPIV3-N antibody and anti-FLAG antibody to detect METTL3. Images were acquired at low magnification using a confocal laser-scanning microscope. Arrows indicate METTL3-positive infected cells, and arrowheads indicate METTL3-positive uninfected cells. Nuclei were counterstained with DAPI. (DOCX) [file ppat.1013755.s003.docx]

**
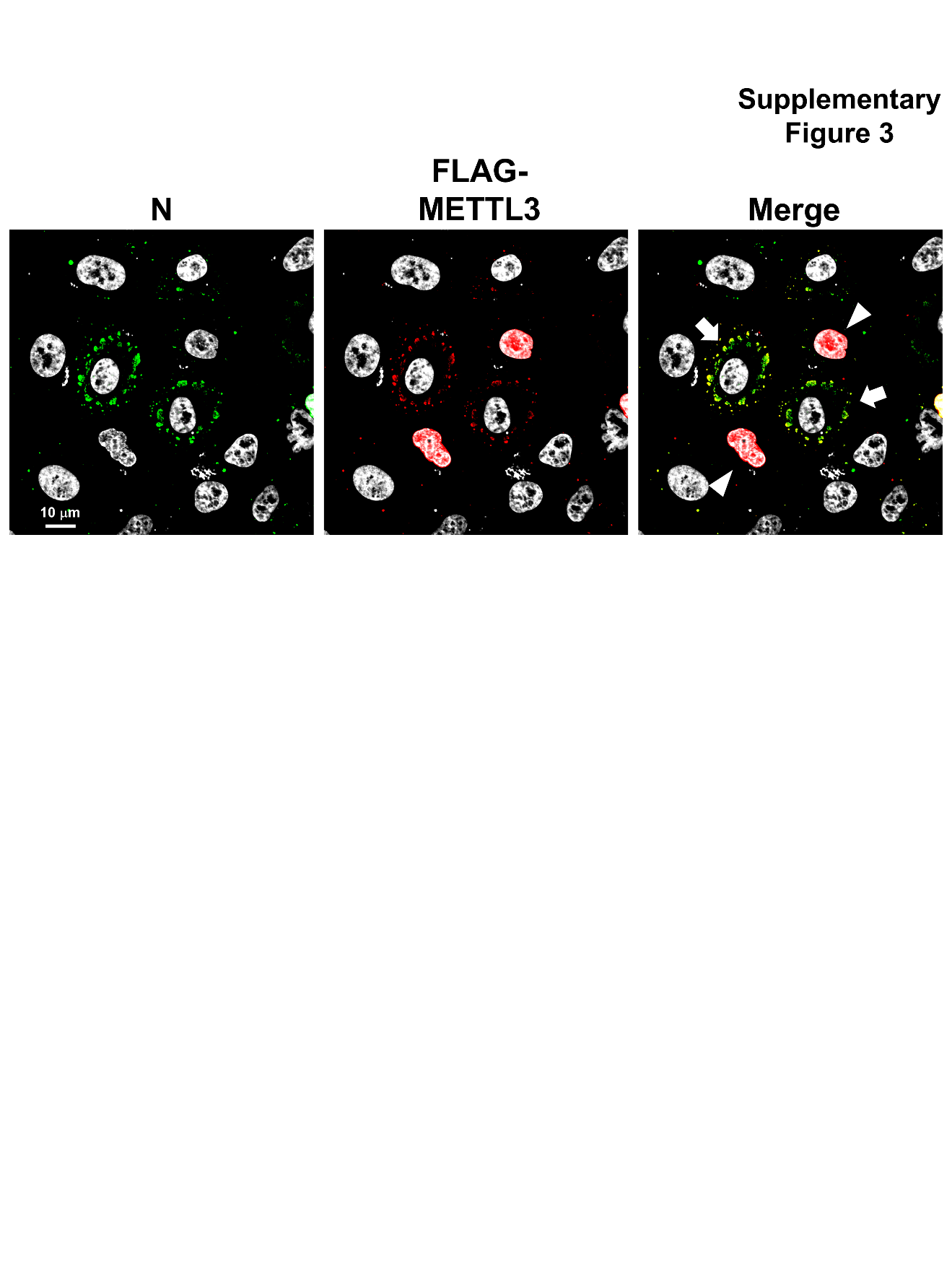
**

**Supplementary Figure 3.** A549 cells were transfected with a FLAG-METTL3 expression plasmid and, at 24 h post-transfection, infected with rBPIV3-EGFP at an MOI of 1. At 48 h post-infection, cells were fixed and co-stained with anti-BPIV3-N antibody and anti-FLAG antibody to detect METTL3. Images were acquired at low magnification using a confocal laser-scanning microscope. Arrows indicate METTL3-positive infected cells, and arrowheads indicate METTL3-positive uninfected cells. Nuclei were counterstained with DAPI.
