# Supplementary material for: Paramyxovirus matrix protein redirects METTL3 for dual regulation of viral replication and immune evasion
Source: PLoS Pathog. 2025 Dec 1;21(12):e1013755. doi: 10.1371/journal.ppat.1013755 (PMC12680350; doi:10.1371/journal.ppat.1013755)
Supplement: S4 Fig — At 48 h post-infection, cells were subjected to UV crosslinking to covalently stabilize interactions between RNA and RNA-binding proteins. Cells were then harvested and lysed. Lysates were immunoprecipitated with anti-dsRNA antibody or control IgG. The immunoprecipitates were analyzed by western blotting using anti-BPIV3-N antibody and anti-FLAG antibody for METTL3 detection. (DOCX) [file ppat.1013755.s004.docx]

**
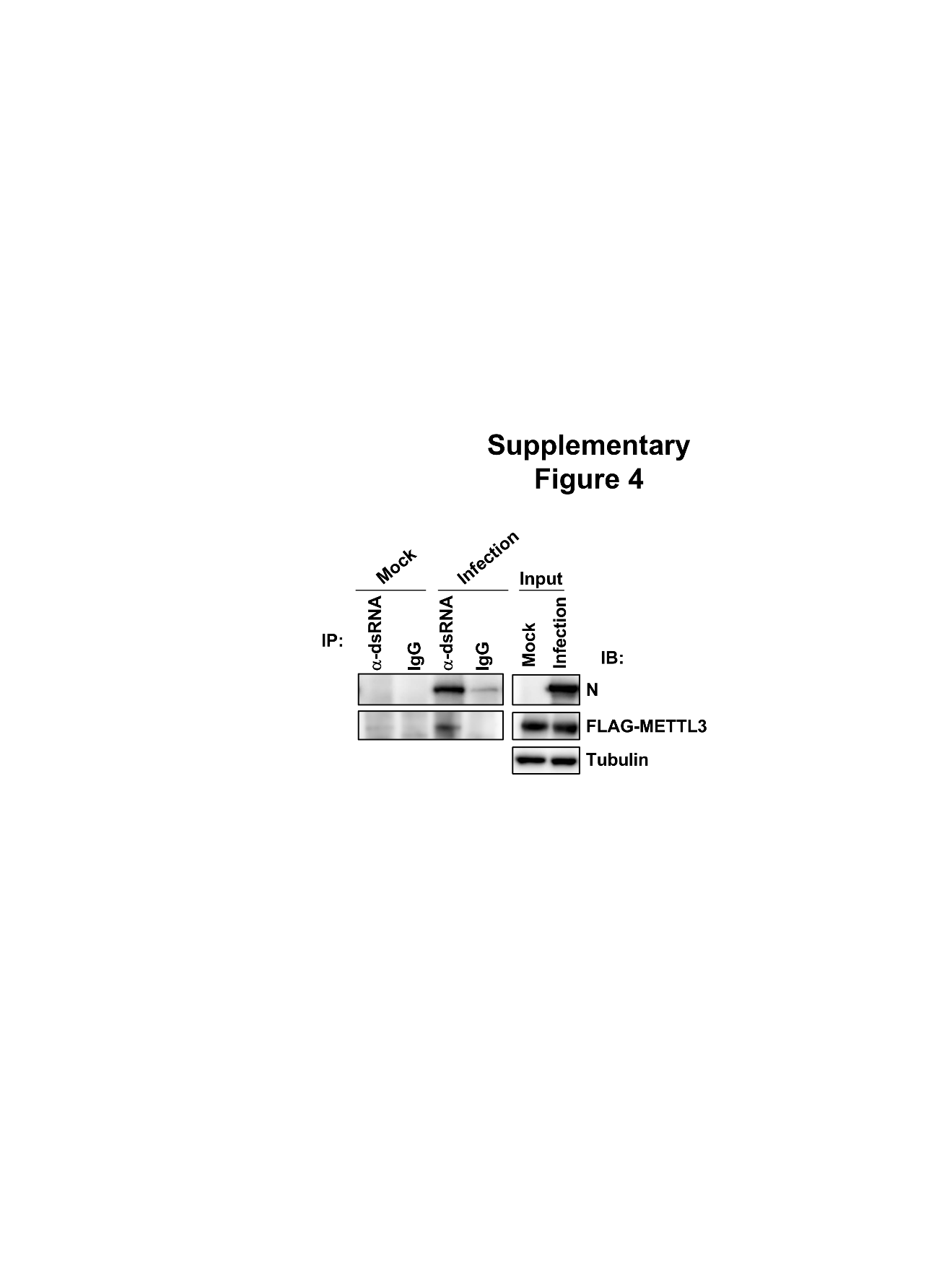
**

**Supplementary Figure 4.** 293T cells were transfected with METTL3 expression plasmid, and at 24 h post-transfection, infected with BPIV3 at an MOI of 1. At 48 h post-infection, cells were subjected to UV crosslinking to covalently stabilize interactions between RNA and RNA-binding proteins. Cells were then harvested and lysed. Lysates were immunoprecipitated with anti-dsRNA antibody or control IgG. The immunoprecipitates were analyzed by western blotting using anti-BPIV3-N antibody and anti-FLAG antibody for METTL3 detection.
