# Supplementary material for: Paramyxovirus matrix protein redirects METTL3 for dual regulation of viral replication and immune evasion
Source: PLoS Pathog. 2025 Dec 1;21(12):e1013755. doi: 10.1371/journal.ppat.1013755 (PMC12680350; doi:10.1371/journal.ppat.1013755)
Supplement: S5 Fig — HeLa cells were singly transfected with METTL3 expression plasmid or cotransfected with METTL3 and N expression plasmids. At 48 h post-transfection, the cells were fixed and stained with anti-FLAG antibody (Ab) only for METTL3 or costained with anti-BPIV3-N Ab and anti-FLAG Ab. (DOCX) [file ppat.1013755.s005.docx]

**
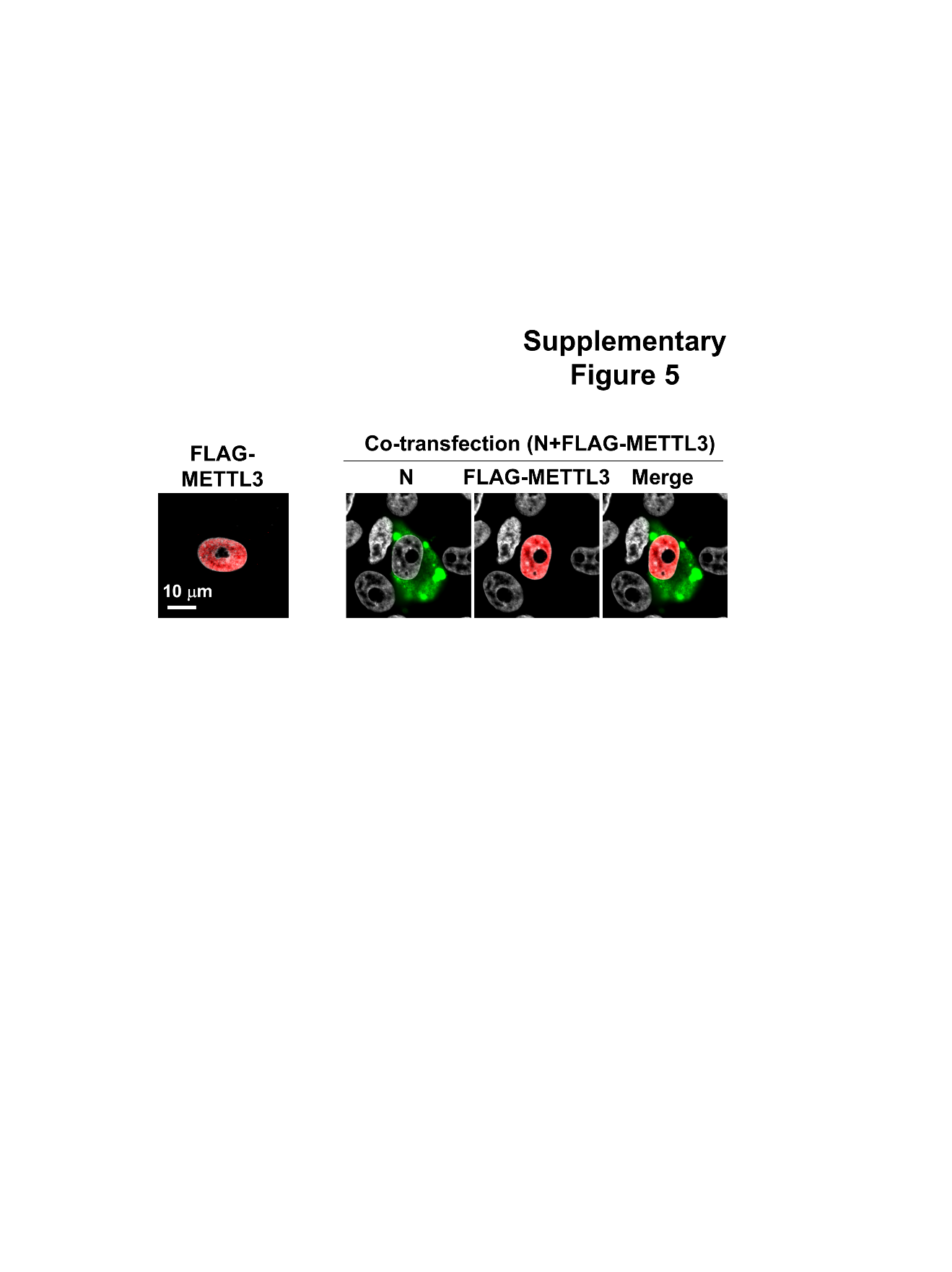
**

**Supplementary Figure 5.** Absence of METTL3 nuclear export during co-expression with viral nucleocapsid protein. HeLa cells were singly transfected with METTL3 expression plasmid or cotransfected with METTL3 and N expression plasmids. At 48 h post-transfection, the cells were fixed and stained with anti-FLAG antibody (Ab) only for METTL3 or costained with anti-BPIV3-N Ab and anti-FLAG Ab.
