# Supplementary material for: Paramyxovirus matrix protein redirects METTL3 for dual regulation of viral replication and immune evasion
Source: PLoS Pathog. 2025 Dec 1;21(12):e1013755. doi: 10.1371/journal.ppat.1013755 (PMC12680350; doi:10.1371/journal.ppat.1013755)
Supplement: S6 Fig — At 24, 48, and 72 hours post-transfection (hpt), cells were fixed and immunostained with an anti-FLAG antibody to detect METTL3 and an anti-M antibody to detect BPIV3-M (A). For classification-based quantification, more than 30 METTL3/M double-positive cells per condition were randomly selected and classified into two localization patterns (“nucleus only” or “cytoplasmic or nucleus”). The number of cells in each pattern was counted, and the results are presented as the percentage of total cells analyzed (B). All experiments were performed independently three times. Asterisks indicate statistically significant differences (*p < 0.05). (DOCX) [file ppat.1013755.s006.docx]

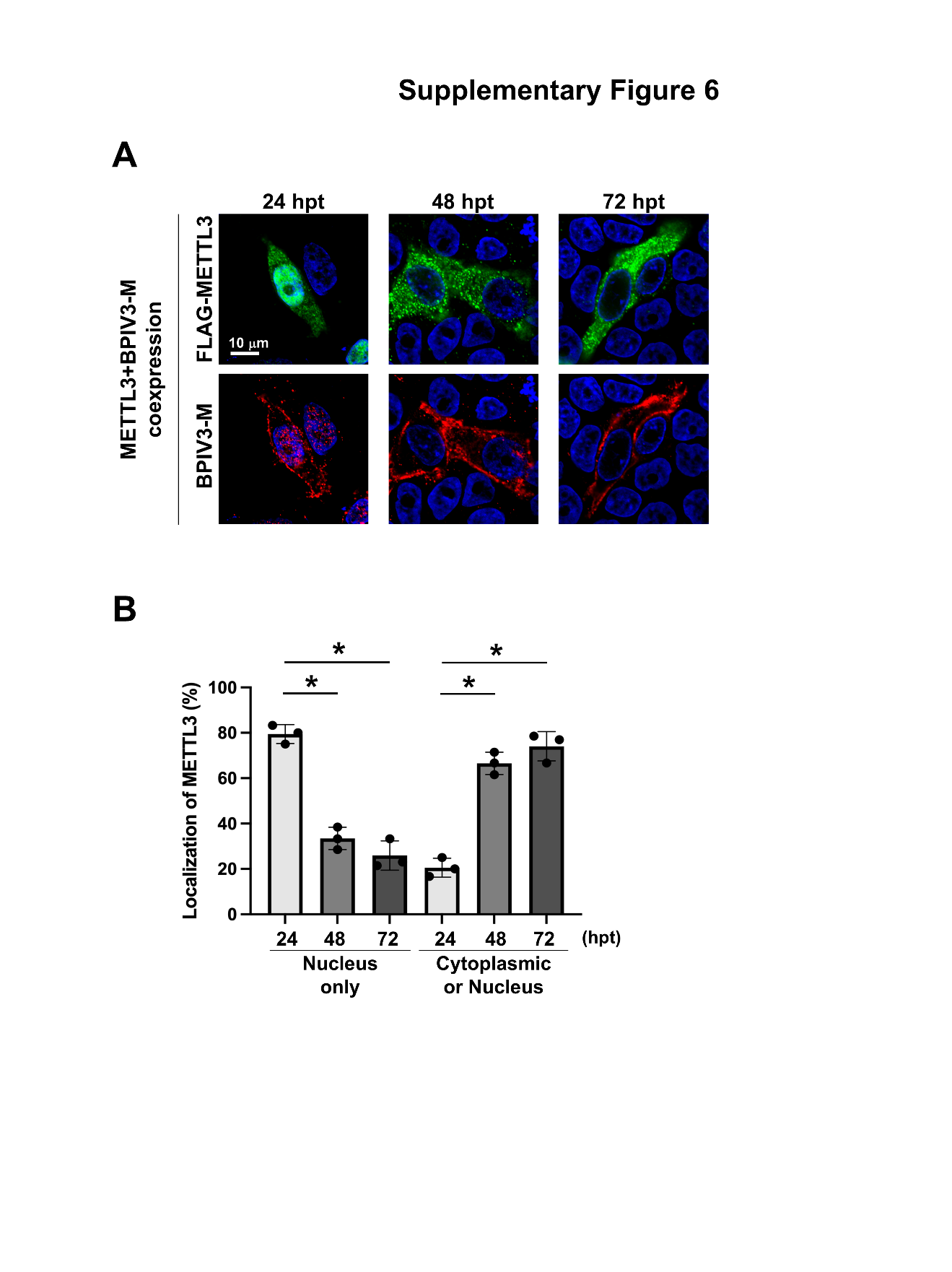


**Supplementary Figure 6.** HeLa cells were cotransfected with METTL3 and BPIV3-M expression plasmids. At 24, 48, and 72 hours post-transfection (hpt), cells were fixed and immunostained with an anti-FLAG antibody to detect METTL3 and an anti-M antibody to detect BPIV3-M (A). For classification-based quantification, more than 30 METTL3/M double-positive cells per condition were randomly selected and classified into two localization patterns (“nucleus only” or “cytoplasmic or nucleus”). The number of cells in each pattern was counted, and the results are presented as the percentage of total cells analyzed (B). All experiments were performed independently three times. Asterisks indicate statistically significant differences (**p* < 0.05).
