# Supplementary material for: Paramyxovirus matrix protein redirects METTL3 for dual regulation of viral replication and immune evasion
Source: PLoS Pathog. 2025 Dec 1;21(12):e1013755. doi: 10.1371/journal.ppat.1013755 (PMC12680350; doi:10.1371/journal.ppat.1013755)
Supplement: S7 Fig — HeLa cells were cotransfected with Sendai virus M (SeV-M), human parainfluenza virus type 3 M (hPIV3-M) +N + P, Nipah virus M (NiV-M), and Measles virus M (MeV-M) +N + P along with METTL3. At 48 h post-transfection, the cells were costained with anti-HA antibody (Ab) for SeV-M, HPIV3-M, and NiV-M, anti-MeV-M Ab and anti-FLAG Ab for METTL3. Nuclei were stained with DAPI (A). For quantification, more than 40 METTL3/M double-positive cells were randomly selected and categorized into two subcellular localization patterns: nucleus only (B) or cytoplasmic or nucleus (C). The number of cells in each category was counted, and the results are presented as the percentage of total cells analyzed. Asterisks indicate statistically significant differences (*p < 0.05). All experiments were performed independently three times. (DOCX) [file ppat.1013755.s007.docx]

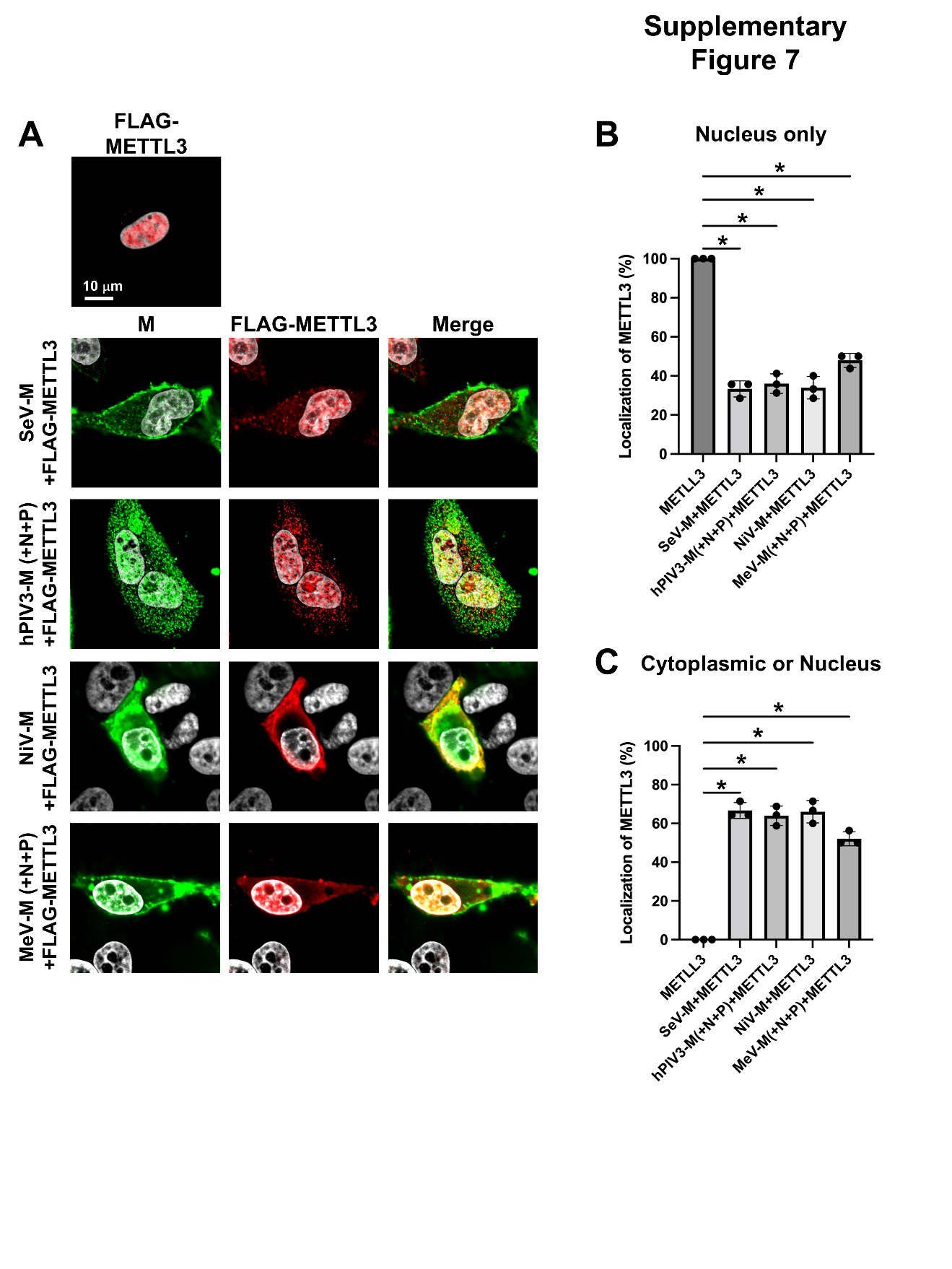


**Supplementary Figure 7.** Common mechanism of M-mediated METTL3 nuclear export among paramyxoviruses. HeLa cells were cotransfected with Sendai virus M (SeV-M), human parainfluenza virus type 3 M (hPIV3-M) +N+P, Nipah virus M (NiV-M), and Measles virus M (MeV-M) +N+P along with METTL3. At 48 h post-transfection, the cells were costained with anti-HA antibody (Ab) for SeV-M, HPIV3-M, and NiV-M, anti-MeV-M Ab and anti-FLAG Ab for METTL3. Nuclei were stained with DAPI (A). For quantification, more than 40 METTL3/M double-positive cells were randomly selected and categorized into two subcellular localization patterns: nucleus only (B) or cytoplasmic or nucleus (C). The number of cells in each category was counted, and the results are presented as the percentage of total cells analyzed. Asterisks indicate statistically significant differences (**p* < 0.05). All experiments were performed independently three times.
