# Supplementary material for: Paramyxovirus matrix protein redirects METTL3 for dual regulation of viral replication and immune evasion
Source: PLoS Pathog. 2025 Dec 1;21(12):e1013755. doi: 10.1371/journal.ppat.1013755 (PMC12680350; doi:10.1371/journal.ppat.1013755)
Supplement: S8 Fig — The m6A probability scores across the open reading frames (ORFs) of all BPIV3 proteins (N, P, M, F, HN, and L) were calculated using the Sequence-based RNA Adenosine Methylation site Predictor (SRAMP). The blue graph represents the m6A probability score at each position, with the horizontal red line indicating the threshold score of 0.5. The analysis revealed several regions with high m6A modification potential, particularly within the N and P gene ORF regions. (DOCX) [file ppat.1013755.s008.docx]

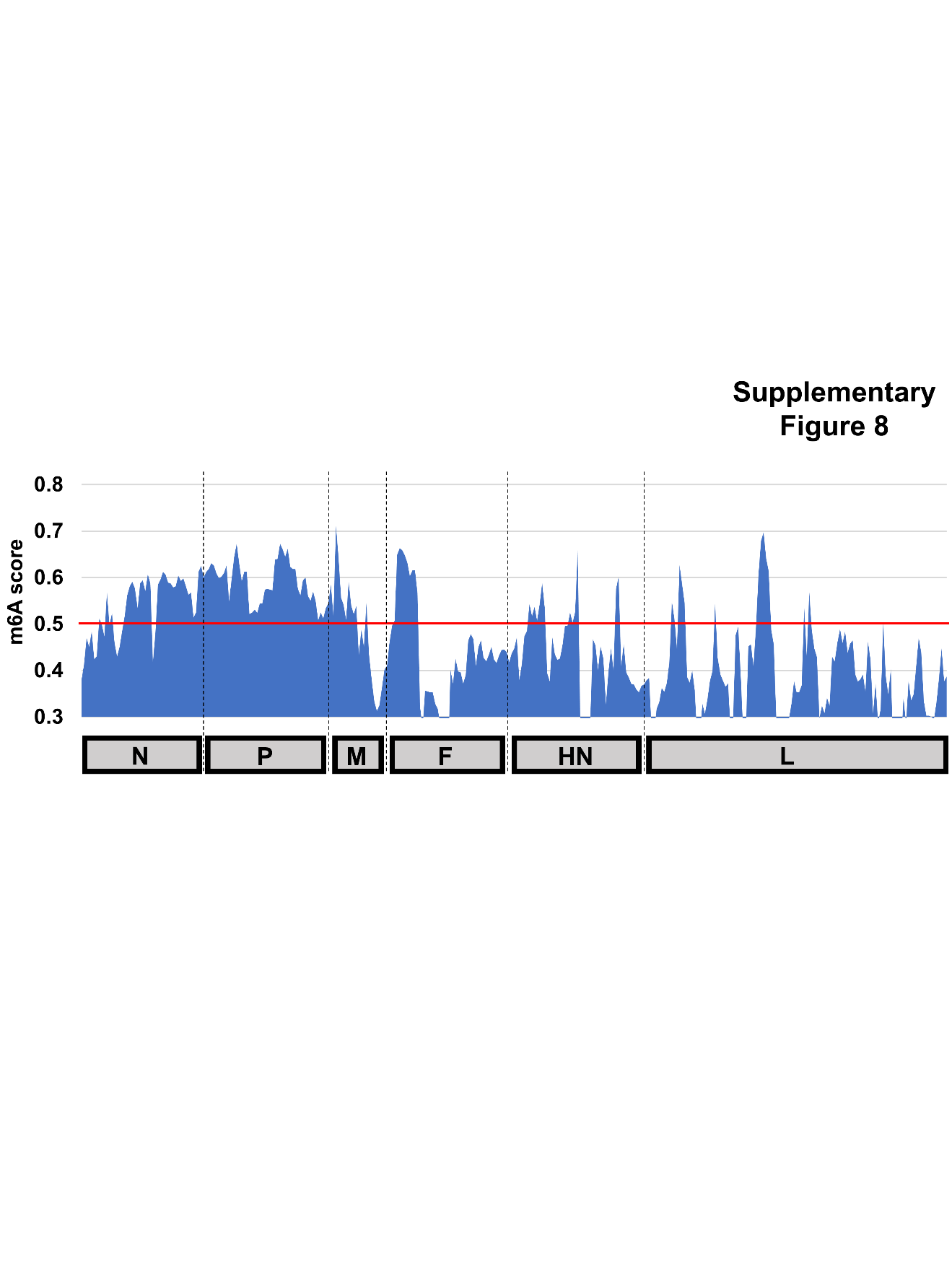


**Supplementary Figure 8.** Prediction of m6A modification sites in BPIV3 protein genes. The m6A probability scores across the open reading frames (ORFs) of all BPIV3 proteins (N, P, M, F, HN, and L) were calculated using the Sequence-based RNA Adenosine Methylation site Predictor (SRAMP). The blue graph represents the m6A probability score at each position, with the horizontal red line indicating the threshold score of 0.5. The analysis revealed several regions with high m6A modification potential, particularly within the N and P gene ORF regions.
