# Supplementary material for: Paramyxovirus matrix protein redirects METTL3 for dual regulation of viral replication and immune evasion
Source: PLoS Pathog. 2025 Dec 1;21(12):e1013755. doi: 10.1371/journal.ppat.1013755 (PMC12680350; doi:10.1371/journal.ppat.1013755)
Supplement: S9 Fig — BHK/T7-9 cells were cotransfected with a BPIV3 minigenome plasmid harboring a T7 promoter and NanoLuc gene together with support plasmids encoding N (wild-type [wt-N] or mutant [N-1372/1427/1443 or N-all]), P, and L proteins. At 48 h post-transfection (hpt), cells were lysed, and NanoLuc luciferase activity was measured. Luciferase activity obtained with mutant N proteins was normalized to that obtained with wt-N. Data are expressed as relative light units (A). A549 and HeLa cells were infected with either wt or N-1372/1427/1443 mutant BPIV3 at an MOI of 5. At 24 h post-infection (hpi), cells were harvested, and N and M proteins were detected by western blotting (B). 293T cells were transfected with either an N expression plasmid or an empty vector. At 24 hpt, cells were infected with either wt BPIV3 or the N-1372/1427/1443 mutant virus at an MOI of 0.1–1. At 24–48 hpi, culture supernatants and cells were collected. Viral titers in the supernatants were determined by the TCID50 assay (C). Total RNA was extracted from infected cells and strand-specific RT-PCR was performed to generate cDNA corresponding to genomic or antigenomic RNA. qPCR using N-specific primers was then carried out to quantify viral genome (D) and antigenome (E) levels. All experiments were performed independently three times. Asterisks indicate statistically significant differences (* p < 0.05); ns, not significant. (DOCX) [file ppat.1013755.s009.docx]

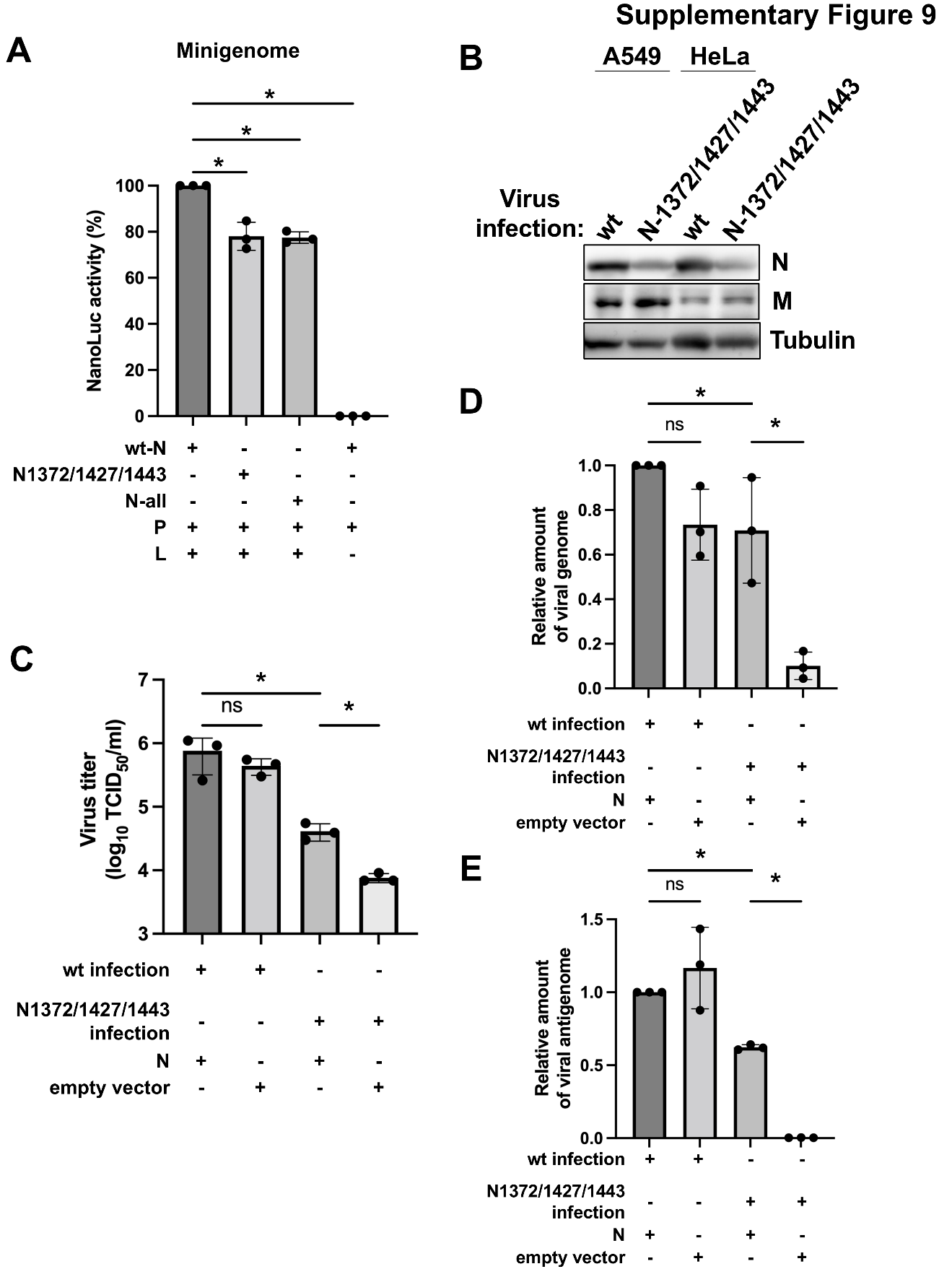


**Supplementary Figure 9.** Effects of N-1372/1427/1443 mutations on viral replication and RNA synthesis. BHK/T7-9 cells were cotransfected with a BPIV3 minigenome plasmid harboring a T7 promoter and NanoLuc gene together with support plasmids encoding N (wild-type [wt-N] or mutant [N-1372/1427/1443 or N-all]), P, and L proteins. At 48 h post-transfection (hpt), cells were lysed, and NanoLuc luciferase activity was measured. Luciferase activity obtained with mutant N proteins was normalized to that obtained with wt-N. Data are expressed as relative light units (A). A549 and HeLa cells were infected with either wt or N-1372/1427/1443 mutant BPIV3 at an MOI of 5. At 24 h post-infection (hpi), cells were harvested, and N and M proteins were detected by western blotting (B). 293T cells were transfected with either an N expression plasmid or an empty vector. At 24 hpt, cells were infected with either wt BPIV3 or the N-1372/1427/1443 mutant virus at an MOI of 0.1–1. At 24–48 hpi, culture supernatants and cells were collected. Viral titers in the supernatants were determined by the TCID_50_ assay (C). Total RNA was extracted from infected cells and strand-specific RT-PCR was performed to generate cDNA corresponding to genomic or antigenomic RNA. qPCR using N-specific primers was then carried out to quantify viral genome (D) and antigenome (E) levels. All experiments were performed independently three times. Asterisks indicate statistically significant differences (* *p* < 0.05); ns, not significant.
