# Supplementary material for: Paramyxovirus matrix protein redirects METTL3 for dual regulation of viral replication and immune evasion
Source: PLoS Pathog. 2025 Dec 1;21(12):e1013755. doi: 10.1371/journal.ppat.1013755 (PMC12680350; doi:10.1371/journal.ppat.1013755)
Supplement: S10 Fig — HeLa cells were transfected with a FLAG-METTL3 expression plasmid and, at 24 hours post-transfection (hpt), infected with either wt-BPIV3 or the M-K258R mutant at an MOI of 1. At 48 hours post-infection (hpi), cells were fixed and co-stained with anti-FLAG antibody for METTL3 and anti-M antibody (A). For classification-based quantification, more than 40 METTL3/M double-positive cells per condition were randomly selected and categorized into two subcellular localization patterns (“nucleus only” or “cytoplasmic or nucleus”). The number of cells in each category was counted, and the results are presented as the percentage of total cells analyzed (B). A549 cells were infected with wt-BPIV3 or the M-K258R mutant at an MOI of 1, and total RNA was extracted from infected cells at 24–48 hpi. After reverse transcription, IFN-β mRNA levels were quantified by qPCR (C). Total RNA extracted from infected or mock-infected cells was subjected to m6A RNA immunoprecipitation using an m6A-specific antibody. IFN-β m6A modification efficiency was determined by qPCR using primer sets targeting m6A sites within IFN-β mRNA, with enriched RNA as the template (D). A549 cells were transfected with a METTL3 expression plasmid or an empty vector, and were infected with BPIV3 at an MOI of 1 at 24 hpt. At 48 hpi, cells were harvested, total RNA was extracted, reverse-transcribed, and IFN-β mRNA levels were quantified by qPCR (E). METTL3-knockdown (KD) or control shRNA HeLa cells were infected with BPIV3 at an MOI of 1. At 48 hpi, cells were processed as in panel E to quantify IFN-β mRNA levels (F). All experiments were performed independently three times. Asterisks represent statistically significant differences (* p < 0.05); ns, not significant. (DOCX) [file ppat.1013755.s010.docx]

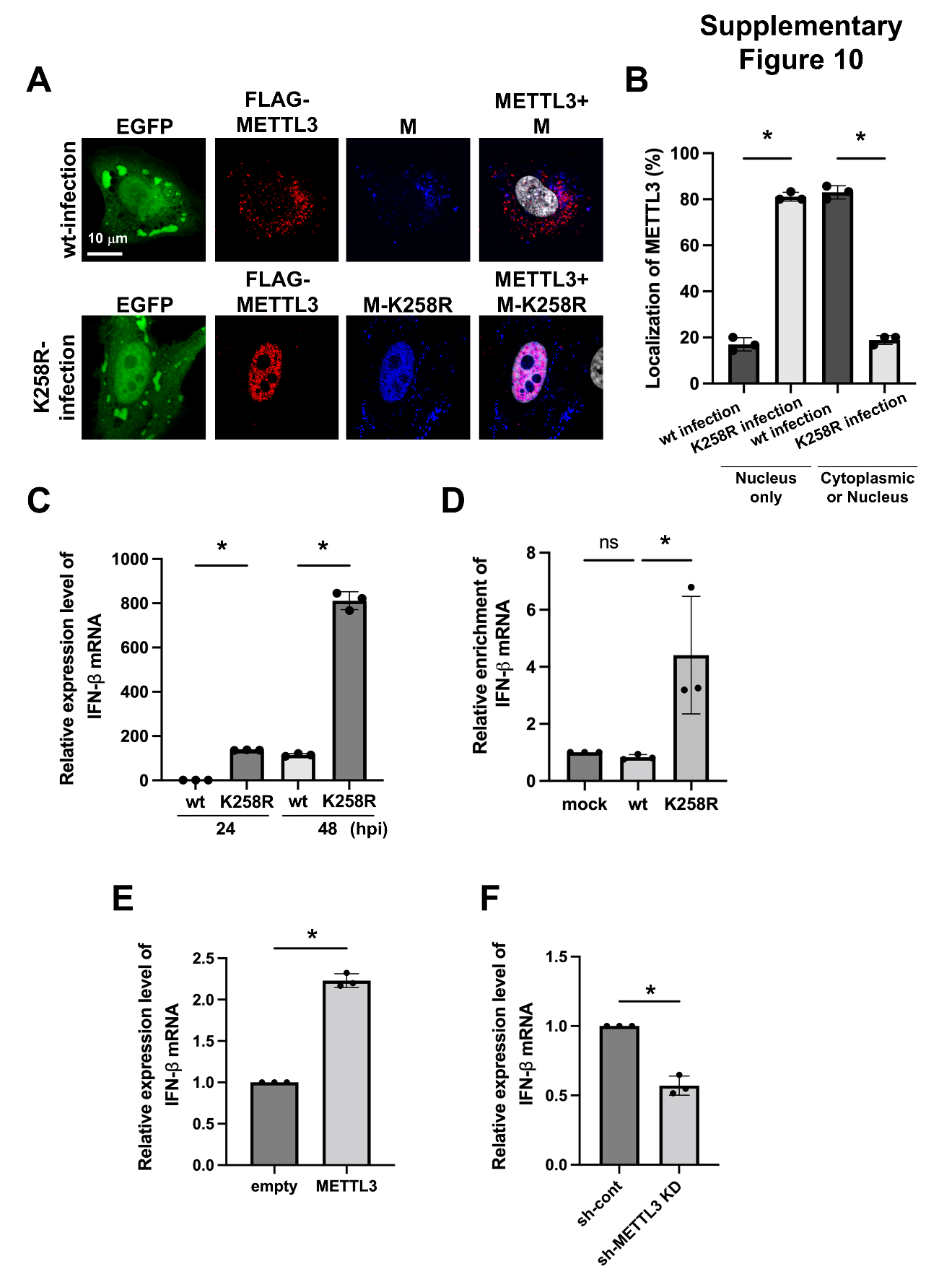


**Supplementary Figure 10.** Impact of METTL3 expression and M-K258R mutation on IFN-β mRNA expression and m6A modification. HeLa cells were transfected with a FLAG-METTL3 expression plasmid and, at 24 hours post-transfection (hpt), infected with either wt-BPIV3 or the M-K258R mutant at an MOI of 1. At 48 hours post-infection (hpi), cells were fixed and co-stained with anti-FLAG antibody for METTL3 and anti-M antibody (A). For classification-based quantification, more than 40 METTL3/M double-positive cells per condition were randomly selected and categorized into two subcellular localization patterns (“nucleus only” or “cytoplasmic or nucleus”). The number of cells in each category was counted, and the results are presented as the percentage of total cells analyzed (B). A549 cells were infected with wt-BPIV3 or the M-K258R mutant at an MOI of 1, and total RNA was extracted from infected cells at 24–48 hpi. After reverse transcription, IFN-β mRNA levels were quantified by qPCR (C). Total RNA extracted from infected or mock-infected cells was subjected to m6A RNA immunoprecipitation using an m6A-specific antibody. IFN-β m6A modification efficiency was determined by qPCR using primer sets targeting m6A sites within IFN-β mRNA, with enriched RNA as the template (D). A549 cells were transfected with a METTL3 expression plasmid or an empty vector, and were infected with BPIV3 at an MOI of 1 at 24 hpt. At 48 hpi, cells were harvested, total RNA was extracted, reverse-transcribed, and IFN-β mRNA levels were quantified by qPCR (E). METTL3-knockdown (KD) or control shRNA HeLa cells were infected with BPIV3 at an MOI of 1. At 48 hpi, cells were processed as in panel E to quantify IFN-β mRNA levels (F). All experiments were performed independently three times. Asterisks represent statistically significant differences (* *p* < 0.05); ns, not significant.
